# Predicting speech from a cortical hierarchy of event-based time scales

**DOI:** 10.1126/sciadv.abi6070

**Published:** 2021-12-03

**Authors:** Lea-Maria Schmitt, Julia Erb, Sarah Tune, Anna U. Rysop, Gesa Hartwigsen, Jonas Obleser

**Affiliations:** 1 Department of Psychology, University of Lübeck, Ratzeburger Allee 160, 23562 Lübeck, Germany.; 2 Center of Brain, Behavior and Metabolism, University of Lübeck, Ratzeburger Allee 160, 23562 Lübeck, Germany.; 3 Lise Meitner Research Group Cognition and Plasticity, Max Planck Institute for Human Cognitive and Brain Sciences, Stephanstraße 1 A, 04103 Leipzig, Germany.

## Abstract

How do predictions in the brain incorporate the temporal unfolding of context in our natural environment? We here provide evidence for a neural coding scheme that sparsely updates contextual representations at the boundary of events. This yields a hierarchical, multilayered organization of predictive language comprehension. Training artificial neural networks to predict the next word in a story at five stacked time scales and then using model-based functional magnetic resonance imaging, we observe an event-based “surprisal hierarchy” evolving along a temporoparietal pathway. Along this hierarchy, surprisal at any given time scale gated bottom-up and top-down connectivity to neighboring time scales. In contrast, surprisal derived from continuously updated context influenced temporoparietal activity only at short time scales. Representing context in the form of increasingly coarse events constitutes a network architecture for making predictions that is both computationally efficient and contextually diverse.

## Teaser

A temporoparietal pathway structures human speech into hierarchically organized events to make predictions on upcoming words.

## INTRODUCTION

While the past predicts the future, not all context that the past provides is equally informative: It might be outdated, contradictory, or even irrelevant. Nevertheless, the brain as a “prediction machine” (*1*) is seemingly equipped with a versatile repertoire of computations to overcome these contextual ambiguities. A prominent example is speech, where a slip of the tongue may render the most recent context uninformative, but we can still predict the next word from its remaining context. At much longer time scales, we can reuse context that suddenly proves informative, as a speaker returns to a topic discussed earlier. Using natural language comprehension as a working model, we here ask: How does the brain dynamically organize, evaluate, and update these complex contextual dependencies over time to make accurate predictions?

A robust principle in the cerebral cortex is the decomposition of temporal context into its constituent time scales along a hierarchy from lower- to higher-order areas, which is evident across species (*2*, *3*), recording modalities (*4*, *5*), sensory modalities (*6*, *7*), and cognitive functions (*8*, *9*). For instance, sensory cortices closely track rapid fluctuations of stimulus features and operate on short time scales [e.g., (*10*)]. By contrast, association cortices integrate stimuli over an extended period and operate on longer time scales [e.g., (*11*)]. Hierarchical specialization has been shown to emerge from structural and functional large-scale connectivity across cortex (*12*, *13*), with connectivity between neighboring areas allowing for efficient mapping between time scales (*14*, *15*).

Conceptually, these hierarchies of “temporal receptive windows” are often subsumed under the framework of predictive coding (*16*): A nested set of time scale–specific generative models informs predictions on upcoming sensory input and is updated on the basis of the actual input (*17*). In particular, context is thought to shape the prediction of incoming stimuli via feedback connections. These connections would link each time scale to its immediate shorter time scale, while the prediction error is propagated forward through the hierarchy (*18*). In line with these accounts of hierarchical predictive coding, feedforward and feedback connections in cortex (*19*, *20*) have been shown to carry prediction errors and predictions (*21*, *22*), respectively.

However, studies on the neural underpinnings of predictive coding have primarily used artificial stimuli of short temporal context [but see (*23*)] and used local versus global violations of expectations, effectively manifesting a two-level cortical hierarchy [but see (*24*)]. We thus lack understanding of whether the hierarchical organization of prediction processes extends to natural environments unfolding their temporal dependencies over a multitude of interrelated time scales.

With respect to functional organization in human cortex, temporoparietal areas are sensitive to a rich set of hierarchies and time scales in speech (*25*, *26*). Most relevant to the present work, semantic context in a spoken story has been shown to map onto a gradient extending from the early auditory cortex representative of words up to the intraparietal sulcus representative of paragraphs (*27*) or the broader storyline in a narrative (*28*). This time scale–specific representation of context is reminiscent of the multilayered generative models proposed to underlie predictive coding (*29*, *30*). Compatible with this notion, previous studies on speech comprehension found evidence for neural coding of prediction errors at the level of syllables (*31*), words (*32*), or discourse (*33*).

However, the interactions between multiple representational time scales of speech in predicting upcoming words remain unclear. Here, we ask whether the temporoparietal processing hierarchy enabling natural speech comprehension is also implicated in evaluating the predictiveness of time scale–specific semantic context and integrating informative context into predictions. We recorded blood oxygen level–dependent (BOLD) responses while participants listened to a narrated story, which provides rich semantic context and captures the full dynamic range of speech predictability. We set out to identify prediction error signaling, which can be formulated as surprisal (*34*). Psycholinguistic surprisal theory states that surprisal is proportional to the cognitive effort required to update a probabilistic prediction when encountering a new word (*35*, *36*), which modulates BOLD activity (*37*) and reading time (*38*).

Here, we focus on two potential computational architectures that could underlie a cortical hierarchy of predictive language comprehension. One attractively simple candidate architecture constructs context representations at all time scales with each upcoming word by replacing outdated context with context just recently encountered. An important implication of such a continuously updating processing hierarchy is that context representations at all time scales are tuned to current processing demands when predicting an upcoming word. In a recent study, Chien and Honey (*39*) showed that a computational model with continuous updates best explained how neural responses to a story rapidly aligned across participants in areas with shorter but only later in areas with longer receptive windows.

A competing candidate architecture is effectively based on the event structure of context, with changes in situational features indicating the end of an event. For example, it is known that scenes in a movie are encoded as event-specific neural responses (*40*) and that more parietal receptive windows represent increasingly coarse events in movies (*41*). The segmentation of context into hierarchical events is implemented via an additional boundary detector that initiates the recombination of an event with preceding events at higher processing stages at an event boundary only. This sparsely updating processing hierarchy calls for fewer updates to context representations, which have been shown to speed up processing (*42*) and allow to draw on semantically more diverse context representations when making predictions. We here hypothesize that such a network architecture is a more appropriate model for prediction processes in the brain.

In the present study, we followed the rationale that neural computations can be inferred by comparing the fit of neural data to outputs from artificial neural networks with different architectures (*43*, *44*). We derived context-specific surprisal associated with each word in the story from single layers of long short-term memory (LSTM)–based language models with either a continuous (*45*) or a sparse updating rule [hierarchical multiscale LSTM (HM-LSTM) (*42*)]. These language models make probabilistic predictions on upcoming words by exploiting representations of context at different time scales with the aim to minimize surprisal and the capability to learn from experience, thereby sharing key features with language prediction in humans (*30*). Furthermore, LSTM models have been shown to effectively explain neural (*46*) and behavioral responses to speech (*47*).

Our results speak to the event-based organization of semantic context as a valid model of predictive processing in the brain. We show that a “surprisal hierarchy” of increasingly coarse event time scales evolves along the temporoparietal pathway, with stronger connectivity to neighboring time scales in states of higher word surprisal. Surprisal derived from continuously updated context had a nonhierarchical effect on temporoparietal activity only at short time scales. Together, these results suggest that representing context in the form of hierarchical events constitutes a network architecture that is computationally efficient while providing a diverse range of context for making predictions.

## RESULTS

Thirty-four participants listened to a 1-hour narrated story while their hemodynamic brain responses were recorded using functional magnetic resonance imaging (fMRI). To emulate a challenging listening scenario, we presented the story against a competing stream of resynthesized natural sounds [for an analysis focusing on cortical representations of acoustics, see (*48*)].

**Fig. 1.**
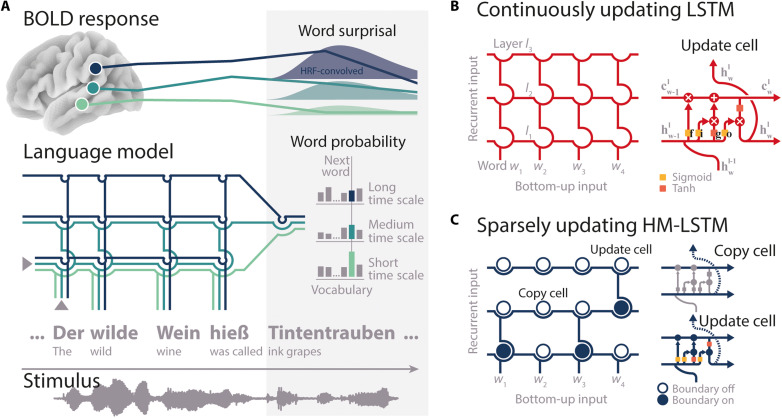
Modeling neural speech prediction with artificial neural networks. (**A**) Bottom: Participants listened to a story (gray waveform) during fMRI. Middle: On the basis of preceding semantic context (“The wild wine was called”), a language model assigned a probability of being the next word to each word in a large vocabulary (gray bars). The probability of the actual next word (“ink grapes”; colored bars) was read out from each layer of the model separately, with higher layers (darker blue colors) accumulating information across longer semantic time scales. Top: Word probabilities were transformed to surprisal, convolved with the hemodynamic response function (HRF, bell shapes), and mapped onto temporoparietal BOLD time series (colored lines). (**B**) Two language models were trained. With each new word-level input, the “continuously updating” LSTM (*45*) combines “old” recurrent long-term ($c_{w-1}^{l}$) and short-term memory states ($h_{w-1}^{l}$) with “new” bottom-up linguistic input ($h_{w}^{l-1}$) at each layer *l*. This allowed information to continuously flow to higher layers with each incoming word. **f**, forget gate; **i**, input gate; **g**, candidate state; **o**, output gate. (**C**) The “sparsely updating” HM-LSTM (*42*) was designed to learn the hierarchical structure of text. An upper layer keeps its representation of context unchanged (copy mechanism) until a boundary indicates the end of an event on the lower layer and information is passed to the upper layer (update mechanism). Networks were unrolled over the sequence of words for illustration only.

The surprisal associated with each word in the story was modeled at multiple time scales of semantic context by two artificial neural networks, an LSTM model with a continuous updating rule and an HM-LSTM model with a sparse updating rule (Fig. 1A). First, we encoded surprisal at multiple time scales into univariate neural responses and fit a gradient to temporoparietal peak locations of time scales. Encoding models were estimated separately per each language model, using ridge regression with fourfold cross-validation. Second, we investigated how surprisal gates the information flow between brain regions sensitive to different time scales.

### Two competing language models of hierarchical speech prediction

We trained two artificial neural networks on more than 130 million words of running text to predict an upcoming word by its preceding semantic context. More specifically, language models consisted of LSTM cells (*45*), which incorporate context that might become relevant at some time (cell state) or that is relevant already to the prediction of the next word (hidden state). By stacking five LSTM layers, our models operated on different time scales of context, with higher layers coding for long-term dependencies between words.

In the continuously updating (or “vanilla”) LSTM, recurrent memory states are updated at each layer with every new bottom-up word input (Fig. 1B). A second model, the sparsely updating HM-LSTM, uses a revised updating rule where information from a lower layer is only fed forward at the end of an event (Fig. 1C). This allows for less frequent updates between layers and stronger separation between contextual information represented at different layers.

### Model-derived metrics of predictiveness at multiple time scales

For each word in the entire presented story (>9000 words), we determined its predictability given the semantic context of the preceding 500 words. Hidden states were combined across layers and mapped to an output module, which denotes the probability of occurring next for every word in a large vocabulary of candidate words. The word with the highest probability was selected as the predicted next word. Overall, the LSTM (proportion correct across words, 0.13) and the HM-LSTM (0.12; fig. S1) were on par in accurately predicting the next word in the story.

To derive the predictability of words based on layer-specific context (or, for our purpose, time scale), we allowed information to freely flow through pretrained networks yet only mapped the hidden state of one layer to the output module by setting all other layer weights to zero. Outputs from these “masked” language models represented the predictiveness of words at one of five time scales.

**Fig. 2.**
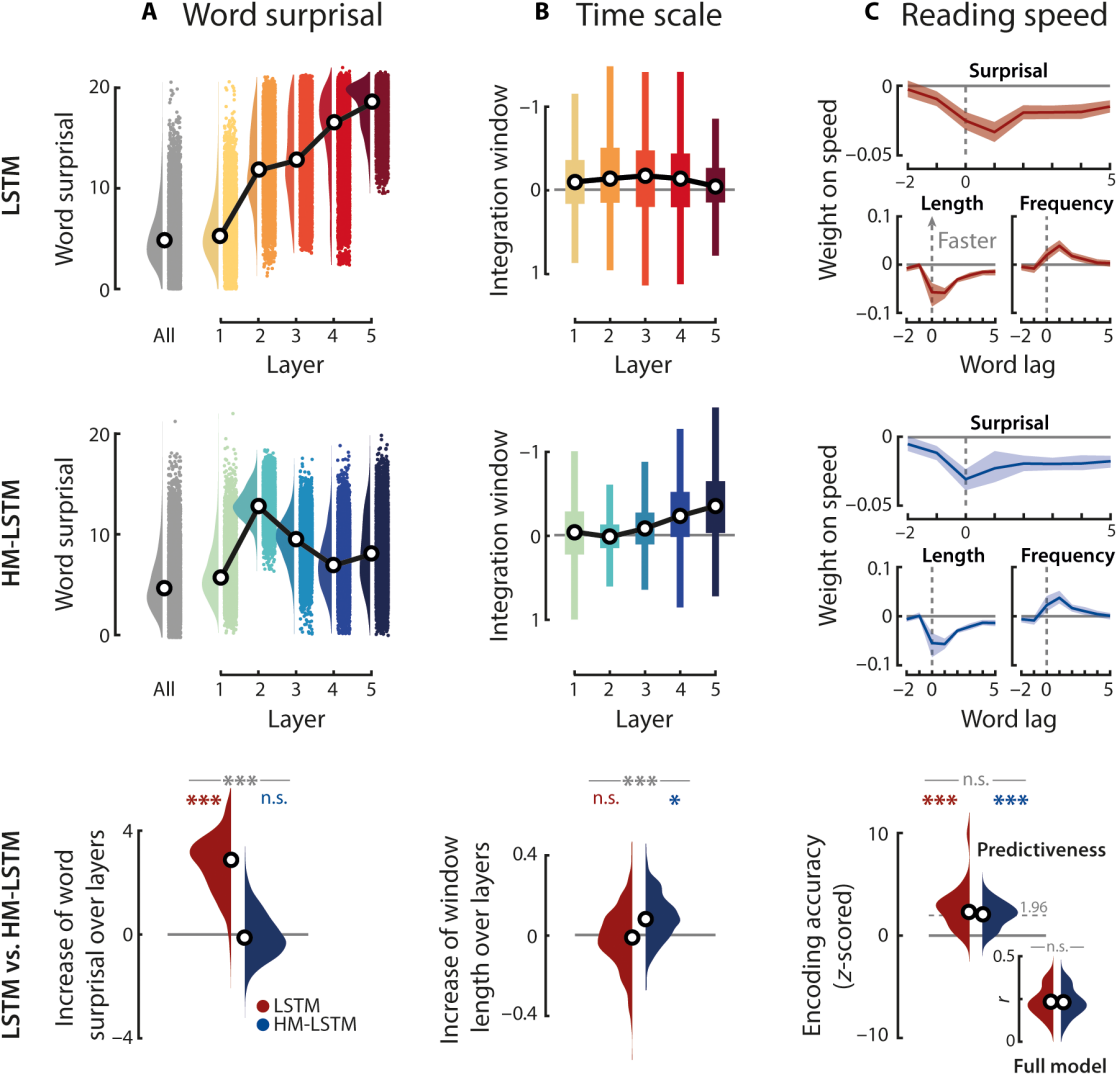
Evaluating model-derived surprisal. (**A**) Word surprisal was derived from full models including all layers (gray distribution) and from single layers of masked models (colored distributions), separately for the LSTM (top) and HM-LSTM (middle); black circles, grand-median surprisal. Linear functions were fit to word surprisal across layers, and resulting slope parameters were compared to empirical null distributions (LSTM, red; HM-LSTM, blue) and between language models (LSTM versus HM-LSTM, gray; bottom). (**B**) Input to models was scrambled at different granularities corresponding to an increase in the length of intact context (i.e., 1 to 256 words). For each layer of models, linear functions were fit to word surprisal across these context windows. A negative slope parameter indicates a stronger benefit (or lower surprisal) from longer context (i.e., larger integration window or time scale). Linear functions fit to integration windows across layers indicate the benefit of higher layers from longer context (bottom). (**C**) Speed in a self-paced reading task was modeled as a function of time-lagged predictiveness and a set of nuisance regressors (i.e., length, frequency, and number of words as well as content versus function words). Weight profiles are shown for surprisal in full models as well as word length and frequency, two factors known to have a major impact on reading speed (*100*) and thereby illustrating the magnitude of the surprisal effect; positive weights indicate an increase in response speed; error bands represent ±SEM. We extracted the encoding accuracy in the self-paced reading task uniquely explained by the predictiveness of context (standardized to scrambled features of predictiveness; bottom); dashed gray line, critical significance level for single participants; inset, nonstandardized encoding accuracies. ***P < 0.001 and *P < 0.05; n.s., not significant.

As the primary metric of predictiveness, we calculated the degree of surprisal associated with the occurrence of a word given its context (i.e., negative logarithm of the probability assigned to the actual next word). The surprisal evoked by an incoming word indexes the amount of information that was not predictable from the context represented at a specific time scale (*35*, *36*). Notably, surprisal was considerably higher at longer time scales in the LSTM (*P* < 0.001, Cohen’s *d* = 2.43; compared to slopes drawn from surprisal shuffled across time scales) but remained stable across time scales in the HM-LSTM (*P* = 0.955, *d* = 0.05; direct comparison of LSTM versus HM-LSTM: *P* < 0.001, *d* = 2.7; Fig. 2A).

To determine the temporal integration window of each time scale, we scrambled input to the network at different granularities corresponding to a binary logarithmic increase in the length of intact context (i.e., 1 to 256 words). The LSTM showed no increase in temporal integration windows at higher layers (LSTM: *P* = 0.219, *d* = 0.11). In contrast, in the HM-LSTM, surprisal decreased more strongly at longer compared to shorter time scales as more intact context became available (HM-LSTM: *P* = 0.027, *d* = 0.73; LSTM versus HM-LSTM: *P* < 0.001, *d* = 0.76; Fig. 2B).

Our secondary metrics expressed the predictability of a word in relation to other words. First, the entropy of the probability distribution over candidate words indicates the difficulty to make a definite prediction on an upcoming word. Second, the dissimilarity of vector representations (or embeddings) coding for the constituent features of the predicted and actual next word (product-moment correlation) indicates conceptual (un-)relatedness.

We derived surprisal, entropy, and dissimilarity associated with single words in the story from masked models at each of five time scales and from “full” models across all time scales, separately for each language model (see fig. S2 for correlations of metrics). All features were convolved with the hemodynamic response function (HRF), and we will collectively refer to them as “features of predictiveness” from here on.

### Higher model-derived surprisal of words slows down reading

To test the behavioral relevance of model-based predictiveness, another 26 participants performed a self-paced reading task where they read the transcribed story word by word on a noncumulative display and pressed a button as soon as they had finished reading. When regressing response speed onto time-lagged features of predictiveness and a set of nuisance regressors (e.g., word length and frequency), we found that, as expected, reading speed slowed down for words determined as more surprising by language models given the full context across all time scales (Fig. 2C).

Furthermore, we predicted response speed on held-out testing data and *z*-scored the resulting encoding accuracy (i.e., product-moment correlation of predicted and actual response speed) to a null distribution drawn from scrambled features of predictiveness while only keeping nuisance regressors intact. This yielded the unique contribution of the predictiveness of words (i.e., surprisal, entropy, and dissimilarity) to reading speed, which was significant for both language models (LSTM: *P* < 0.001, *d* = 1.51; HM-LSTM: *P* < 0.001, *d* = 1.64; LSTM versus HM-LSTM: *P* = 0.975, *d* = 0.35). Together, these findings suggest that both language models picked up on processes of speech prediction that shape behavior.

### Selecting temporoparietal regions of interest involved in speech processing

We hypothesized that the speech prediction hierarchy is represented as a gradient along the temporoparietal pathway. This rather coarse region of interest (ROI) was further refined to only include regions implicated in processing of the listening task.

**Fig. 3.**
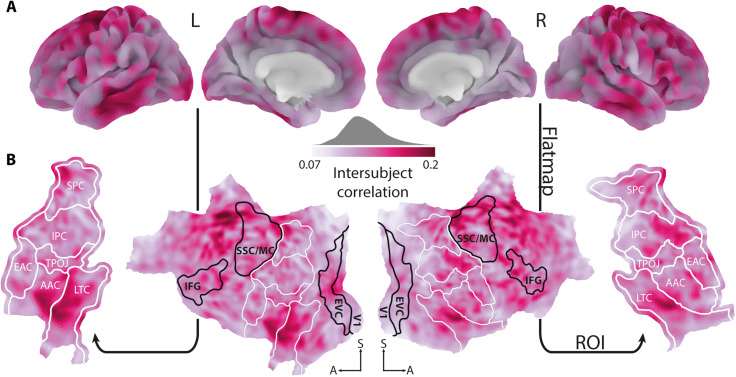
Selection of ROIs. (**A**) When listening to a story against background noise, pairwise intersubject correlations showed stronger synchronization of BOLD activity in cortical areas implicated in the language network. (**B**) The cortical surface was flattened. All temporal and parietal parcels (*51*) highlighted by white outlines were included as ROIs in the following analyses. Black outlined parcels serve as reference point only. EAC, early auditory cortex; AAC, auditory association cortex; LTC, lateral temporal cortex; TPOJ, temporo-parieto-occipital junction; IPC, inferior parietal cortex; SPC, superior parietal cortex; V1, primary auditory cortex; EVC, early visual cortex; SSC/MC, somatosensory and motor cortex; L, left; R, right. Maps were smoothed with an 8-mm full width at half maximum (FWHM) Gaussian kernel for illustration only.

To this aim, we calculated pairwise intersubject correlations (*49*), which revealed consistent cortical activity across participants in a broad bilateral language network. Responses were most prominently shared in auditory association cortex and lateral temporal cortex as well as premotor cortex, paracentral lobule, and mid cingulate cortex (Fig. 3A). Crucially, as sound textures presented in the competing stream were randomly ordered across participants, this approach allowed us to extract shared responses specific to the speech stream.

The context representations necessary to make predictions are thought to be located in temporal and parietal cortex regions, while most other regions showing increased intersubject correlations are thought to be implicated in networks of cognitive control and action (*50*). Therefore, we limited all further analyses to those temporal and parietal parcels (*51*), in which more than 80% of vertices yielded a significant intersubject correlation. We tested for significance by ranking the median group-level intersubject correlation of single vertices against a bootstrapped null distribution [*P* < 0.01, adjusted for false discovery rate (FDR)]. The cortical sheet of the six parcels determined as ROIs was flattened, resulting in a two-dimensional plane spanned by an anterior-posterior and inferior-superior axis (Fig. 3B). We expected gradients of speech prediction to unfold along the inferior-superior axis, that is, from temporal to parietal areas.

### Differential tuning to continuously versus sparsely updated time scales of surprisal in temporoparietal cortex

In an encoding analysis, we regressed hemodynamic responses of single vertices in the temporoparietal ROI onto model-based features of predictiveness (i.e., surprisal, entropy, and dissimilarity at single time scales and for full models) and a set of acoustic and linguistic nuisance regressors. For each language model, we extracted five temporoparietal maps showing the time scale–specific regression weights of surprisal.

**Fig. 4.**
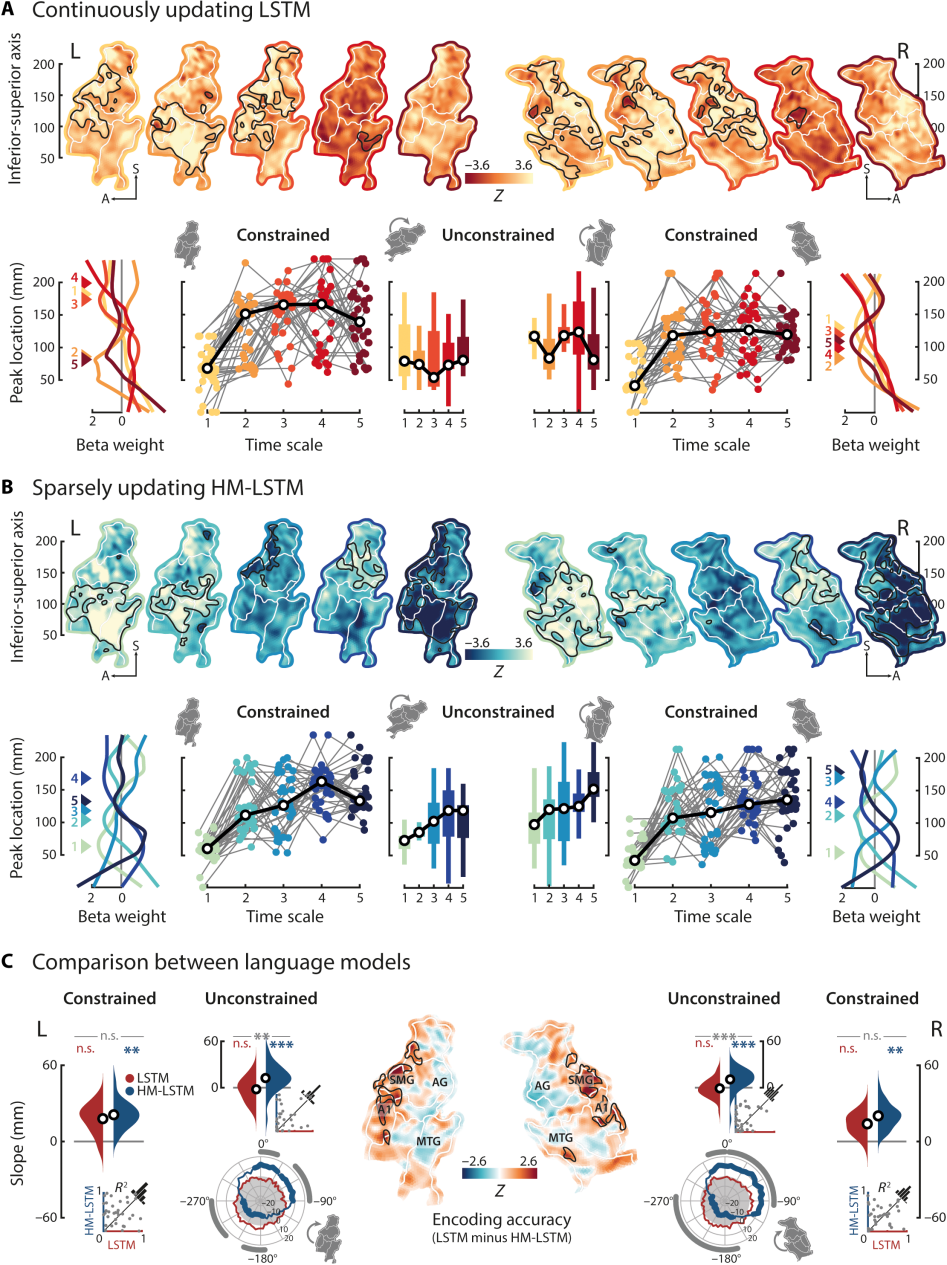
Encoding surprisal at multiple time scales. (**A**) Top row: Temporoparietal weight maps of LSTM-derived surprisal at each time scale were tested against zero; positive *z* values indicate increased BOLD activity in response to more surprising words; black outlines, significant clusters; white outlines, parcels; colored outlines, short (light) to long (dark) time scales, separately for the left and right hemispheres. Bottom row: Time scale–specific peak coordinates were determined along the inferior-superior axis (colored triangles numbered according to time scale), shown for grand-average weight profiles. Testing for a processing hierarchy along the dorsal stream, time scales were constrained to peak superior to the first time scale; colored dots, single-subject peak coordinates; black circles, grand-median peak coordinates. In the unconstrained approach, time scales were allowed to peak at any location, and maps were rotated around the inferior-superior axis (shown for −45°) to test for the spatial specificity of the effect. (**B**) Same as above but for the HM-LSTM. (**C**) Linear functions were fit to peak coordinates across time scales, and resulting slope parameters were compared to empirical null distributions (LSTM, red; HM-LSTM, blue) and between language models (LSTM versus HM-LSTM, gray); black circles, grand-average slope parameters; insets, coefficients of determination for single-subject fits. In addition, we tested for slope effects around the full circle (rose plots); white areas indicate positive slope parameters; fat colored lines, significant slope clusters of single language models; fat gray lines, significant clusters of slope differences between language models. Maps of encoding accuracies were *z*-scored to null distributions drawn from scrambled features of predictiveness and compared between language models. SMG, supramarginal gyrus; AG, angular gyrus; A1, primary auditory cortex; MTG, middle temporal gyrus. Maps were smoothed with an 8-mm FWHM Gaussian kernel for illustration only. **P < 0.01 and ***P < 0.001.

When performing spatial clustering on these weight maps (*P*_vertex_ and *P*_cluster_ < 0.05; compared to scrambled surprisal by means of a cluster-based permutation test), we found large positive clusters in both hemispheres for surprisal at shorter time scales of the LSTM (Fig. 4A, yellow outlines) but, if at all, only focal clusters at longer time scales (Fig. 4A, red outlines). Hence, temporoparietal activity primarily increased in response to words that were less predictable by the context provided at shorter, continuously updated time scales. In contrast, we found clusters of distinct polarity, location, and extent for surprisal at all time scales of the HM-LSTM (Fig. 4B). This suggests that even longer time scales had the potency to modulate temporoparietal activity when they were sparsely updated.

### Sparsely updated time scales of surprisal evolve along a temporoparietal processing hierarchy

To probe the organization of surprisal at different time scales along a temporoparietal gradient, we collapsed across the anterior-posterior axis of single weight maps and selected the local maximum with the largest positive value on the inferior-superior axis. Fitting a linear function to these peak coordinates of time scales, we found flat slope parameters indicating random ordering of LSTM time scales in both hemispheres (left: *P* = 0.458, *d* = −0.15; right: *P* = 0.716, *d* = −0.07; compared to slopes drawn from coordinates scrambled across time scales; Fig. 4C). Conversely, we found steep positive slopes for the HM-LSTM in both hemispheres (left: *P* < 0.001, *d* = 0.72; right: *P* < 0.001, *d* = 0.75; Fig. 4C), reflecting the representation of surprisal at longer time scales in more parietal regions. The grand-average slope of the HM-LSTM in the left hemisphere indicates that surprisal at a sparsely updated time scale is represented 12 mm superior to its directly preceding time scale along the unfolded temporoparietal surface. Most relevant, this finding was underpinned by a significant difference of slope parameters between the LSTM and HM-LSTM (left: *P* = 0.005, *d* = 0.9; right: *P* < 0.001, *d* = 0.89; Fig. 4C), demonstrating a temporoparietal processing hierarchy of word surprisal that preferably operates on sparsely updated time scales.

The absence of a gradient for surprisal at continuously updated time scales was corroborated when specifically targeting the dorsal processing stream. To this aim, we confined surprisal at the first time scale to peak in temporal regions and surprisal at all other time scales to peak superior to the first time scale in more parietal regions. Slope effects along the dorsal stream largely complied with those found in the unconstrained approach (LSTM, all *P* ≥ 0.134; HM-LSTM, all *P* ≤ 0.006; LSTM versus HM-LSTM, all *P* ≥ 0.103), thereby ruling out the possibility that the presence of a competing ventral stream obscured the consistent ordering of time scales.

In addition, rotating weight maps around the full circle in steps of 5° before collapsing across the first dimension showed that surprisal at sparsely updated time scales was processed along a broad gradient, roughly covering rotations from 0° to −90° in both hemispheres (*P*_vertex_ and *P*_cluster_ < 0.05; compared to scrambled surprisal by means of a cluster-based permutation test). No effect was found for the LSTM.

In a complementary decoding approach (see text S7), we reconstructed surprisal at the five time scales from patterns of neural activity in single ROIs (i.e., temporoparietal parcels). Contrasting decoding accuracies between language models, left-hemispheric early auditory cortex contained more information about surprisal at medium, sparsely updated time scales, whereas inferior and superior parietal cortex preferentially represented surprisal at long, sparsely updated time scales (fig. S3). This finding converges with the organization of the gradient described earlier for sparsely updated but not continuously updated time scales of surprisal.

Unlike for the sparsely updated time scales of surprisal, neither the time scales of entropy (all *P* ≥ 0.583) nor dissimilarity (all *P* ≥ 0.623) organized along a dorsal gradient (figs. S4 and S5). Furthermore, effects of HM-LSTM time scale surprisal were dissociable from a simple measure of semantic incongruence between words in the story and their preceding context at five time scales logarithmically increasing in length (all *P* ≥ 0.5; product-moment correlation of target and average context embedding). This highlights the specificity of the observed gradient to prediction processes in general and word surprisal in particular.

To determine the contribution of predictiveness to overall encoding accuracy on held-out data, we *z*-scored accuracies relative to null distributions drawn from scrambled features of predictiveness while keeping additional (spectrotemporal) acoustic and linguistic nuisance regressors intact (for encoding accuracies of these other regressors, see fig. S6). The LSTM produced, in comparison to the HM-LSTM, better predictions in early auditory cortex and supramarginal gyrus (*P*_vertex_ and *P*_cluster_ < 0.05; cluster-based permutation paired-sample *t* test; Fig. 4C). On the other hand, predictions of the HM-LSTM seemed slightly more accurate than for the LSTM along the middle temporal gyrus, temporo-parieto-occipital junction, and angular gyrus, although not statistically significant. Taking into account the broad clusters found earlier specifically for surprisal at shorter (but not longer) LSTM time scales, this underscores the notion that surprisal at continuously updated time scales takes full effect only in earlier processing stages, whereas the sparsely updating processing hierarchy evolves to more posterior parietal regions.

### Surprisal at sparsely updated time scales gates connectivity along the processing hierarchy

After establishing the temporoparietal processing hierarchy, we examined the modulatory effect of surprisal on connectivity between peak locations of time scales taken from the encoding analysis. Following the assumptions of predictive coding (*16*), the bottom-up information flow from a brain area representative of a shorter time scale to its immediately neighboring, longer time scale should increase when the prediction error at the shorter time scale is higher, thereby initiating updates to predictions at the longer time scale. In turn, higher time scale–specific surprisal should increase the top-down information flow to the immediately shorter time scale, thereby indicating the backward-pass of updated predictions. Such a surprisal-gated hierarchy is thought to allow for efficient mapping between time scales.

**Fig. 5.**
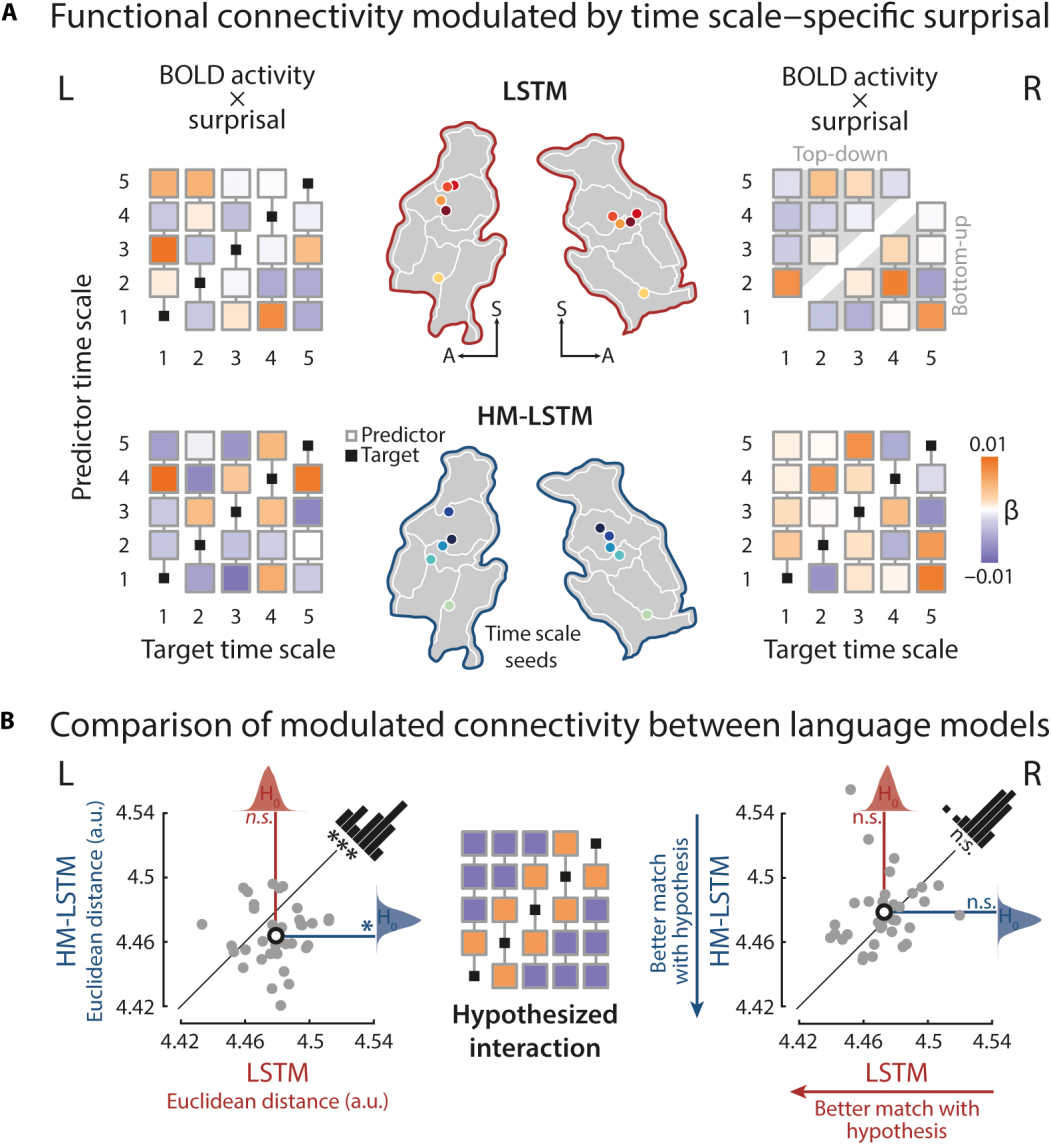
Surprisal-dependent modulation of effective connectivity. (**A**) A sphere of 6 mm was centered on median peak locations of the time scales of surprisal as defined in the encoding analysis (colored circles on temporoparietal maps), and BOLD responses were averaged within these time scale seeds. BOLD time series at one (target) time scale were regressed onto PPIs of all other (predictor) time scales (i.e., pointwise product of time scale–specific BOLD and surprisal time series). For each target seed, we added a column vector of time scale–specific predictor weights to a five-by-five matrix with an empty main diagonal. Matrices were created separately for each language model (top, LSTM; bottom, HM-LSTM) and hemisphere. The upper triangle of a matrix indicates top-down, and the lower triangle indicates bottom-up information flow. (**B**) A hypothesized matrix of PPIs was created, with positive weights on diagonals below and above the main diagonal (orange squares), indicating increased connectivity between surprisal at neighboring time scales when surprisal is high. The Euclidean distance between observed and hypothesized matrices was compared to null distributions of distances drawn from target time scales shifted in time (LSTM, red density plot; HM-LSTM, blue) and between language models (LSTM versus HM-LSTM, level of significance shown in black), separately for each hemisphere; gray dots, distances of single participants; black circles, mean distances. *P < 0.05 and ***P < 0.001. a.u., arbitrary units.

To this aim, we created psychophysiological interactions (PPIs) (*52*) between the BOLD response at the peak location of one time scale and word surprisal at the same time scale. The BOLD response at the peak location of each (target) time scale was regressed onto the PPIs of all other (predictor) time scales (Fig. 5A). A positive weight indicates increased coupling between two peak locations when surprisal at the predictor time scale is high.

We hypothesized that coupling between brain regions representing surprisal at two neighboring time scales increases when one time scale becomes unpredictive. Numerically, this can be expressed by setting the weights of neighboring time scales to 1 and all other predictor weights to −1 (Fig. 5B). This hypothesized pattern of weights was not matched by the weights observed for the LSTM (left: *P* = 0.83, *d* = 0.27; right: *P* = 0.348, *d* = 0.1; Euclidean distance compared to null distributions drawn from target BOLD activity shifted in time), which was expected given that surprisal at the continuously updated time scales was not organized along a gradient in the first place. Critically, for surprisal at sparsely updated time scales of the HM-LSTM, surprisal-modulated connectivity in the left hemisphere not only matched our hypothesis (left: *P* = 0.032, *d* = 0.5; right: *P* = 0.853, *d* = 0.23) but also matched our hypothesis better than the LSTM (left: *P* = 0.001, *d* = 0.79; right: *P* = 0.902, *d* = 0.3).

To specify the overall directionality of information flow, we separately averaged the weights of top-down modulations (i.e., predictor weights of surprisal at time scales longer than respective target time scales) and bottom-up modulations. We found no difference between the modulatory strength of these top-down and bottom-up connections (LSTM left: *P* = 0.184, *d* = 0.23; LSTM right: *P* = 0.839, *d* = 0.4; HM-LSTM left: *P* = 0.408, *d* = 0.4; HM-LSTM right: *P* = 0.367, *d* = 0.16).

## DISCUSSION

How are the complex temporal dependencies underlying natural speech processed in the brain to inform predictions of upcoming speech? In the present study, we simulated these prediction processes in two language models (LSTM versus HM-LSTM), which critically differed in how often semantic context representations are updated at multiple, hierarchically organized time scales.

Surprisal as derived from both language models modulated reading times in the behavioral reading task to a similar degree. However, hemodynamic brain responses to surprisal during the listening task differed between models: In line with our initial hypothesis, temporoparietal regions hierarchically encoded the (sparsely updated) event-based surprisal provided by the layers of the HM-LSTM, with surprisal at longer time scales represented in inferior parietal regions. Moreover, higher time scale–specific surprisal based on the HM-LSTM increased connectivity from receptive windows of surprisal at a given time scale to their immediately neighboring (shorter or longer) time scales.

Together, these results provide evidence for the neurobiological parsimony of an event-based processing hierarchy. In the present data, this was expressed in the simultaneous neural representation of surprisal at multiple time scales and in surprisal dynamically gating the connectivity between these time scale–specific receptive windows.

### The event-based organization of context as a foundation for language prediction

The spatial organization of time scale–specific receptive windows of surprisal observed in the present study converges with previous results, where bilateral primary auditory cortex coded for relatively shorter time scales (e.g., words) and inferior parietal cortex coded for longer time scales (e.g., paragraphs) (*27*). Critically, this spatial overlap was found despite targeting different aspects of speech processing.

In our study, neural responses were expressed as a function of time scale–specific word surprisal, a proxy tapping into prediction processes. In other studies, receptive windows were based on the (in-)consistency of neural activity across participants in response to speech input at varying time scales (*53*), which is typically linked to working memory formation. This implies that the same neural system most likely fulfils distinct functions: Temporal receptive windows have been suggested to store time scale–specific context in working memory and, in parallel, exploit this context to process information in the present (*9*). In line with this theoretical account, our results suggest that time scale–specific memory representations serve as the basis for the generative models shaping predictions of upcoming speech.

Here, the observed temporoparietal gradient of surprisal at sparsely updated representations of context is specifically well in line with accounts of neural event segmentation (*54*, *55*) and with the notion of hierarchical multiscale network architectures more generally (here, HM-LSTM). Taking a sentence from our listening task as an example, “The wild wine was called ink grapes” represents a brief event where the narrator describes how the bluish black of the grapes in a backyard reminded her of the color of the night. At the same time, the sentence is preceded by a larger event of the author walking the streets on the way to her parents’ house. Both events can be combined into an even larger event of the author visiting her Romanian hometown.

Notably, the events modeled by the HM-LSTM do not necessarily concur with human event annotations and are not fully independent of previous events because of a leaky network architecture. Notwithstanding these limitations, we can draw an analogy between the HM-LSTM architecture and neural event segmentation in two decisive points. First, the boundary detector allows revealing an event structure of context, similar to an increase in neural activity indexing prediction errors at event boundaries (*56*, *57*). Second, the sparse updates to higher processing stages at event boundaries allow retaining multiple, stable context representations in memory, similar to temporo-parieto-occipital receptive windows reflecting the hierarchical event structure during movie watching (*41*). We directly tie in with this result by showing that this hierarchical, event-based context enables neural prediction processes.

What are the computational and mechanistic implications of this contextual architecture for the prediction of speech? Somewhat paradoxically, event models have been referred to as “an added burden for an organism” (*58*). This argument is certainly plausible with regard to the size of the parameter space, which increases in an artificial or, likewise, biological neural network by introducing an additional boundary detector. At the expense of model parsimony, however, such an event-based network allows for fewer updates in comparison to continuously updating networks such as the LSTM, where each new input to the model elicits computationally complex updates to all time scales.

The trade-off between computational costs of boundary detector and update frequency is nicely illustrated by the fact that the sparsely updating HM-LSTM is considerably faster in making predictions than the continuously updating LSTM (*42*). Thus, from a functional perspective, keeping layered representations of multiple events in memory allows to efficiently draw on diverse information to make predictions on upcoming speech.

### Context-dependent surprisal as a gating mechanism for predictions and prediction errors

The hallmark of prediction processes in our data is the increase in reading times and neural activity observed in response to more surprising input (*21*, *59*). There are different computational ways in which this “expectation suppression” (*60*) can be realized, namely integration difficulty, neural sharpening, and predictive coding.

One take on expectation suppression is that surprising sensory input is more difficult to integrate into already existing representations of context because it conveys a relatively larger amount of new information (*61*). The architecture of the sparsely updating HM-LSTM dictates that new information is integrated into time scale–specific representations only at event boundaries, thereby suggesting that integration difficulty should arise primarily on an event-by-event basis. As surprisal varies on a word-by-word basis, it is less likely that integration difficulty accounts for our effects of event-based surprisal. However, accounts of integration difficulty and predictive processing are not mutually exclusive. The other two accounts both assume that expectation suppression is indicative of prediction processes but differ in how these processes are thought to be implemented in the brain.

Sharpening accounts argue that unexpected components of sensory input are suppressed via feedback predictions (*62*), while accounts of predictive coding (*16*, *63*) suggest that the brain filters out (or “dampens”) expected components of sensory input. Both accounts assume that predictive processes lead to an overall decrease in neural activity to incoming stimuli. The similarity between hypothesized response patterns makes it notoriously hard to disentangle those accounts (*32*).

Notably, however, a distinguishing feature of predictive coding is the specificity of feedforward prediction error signals, which can be captured by modeling effective connectivity between receptive windows of time scale–specific surprisal. In agreement with the hierarchical information flow laid out in predictive coding (*16*), surprisal in our study modulated connectivity via bidirectional links between neighboring receptive windows of longer and shorter event-based time scales in the left hemisphere (Fig. 5).

Surprisal in the event-based artificial neural network was modeled as the amount of information an input word conveys that cannot be explained away by the context (or generative model) represented at a specific time scale. Therefore, the increase in feedforward connectivity in response to higher surprisal precisely aligns with the concept of prediction errors in predictive coding (*63*).

In addition, the increase in feedback connectivity in response to higher surprisal accords with an electrocorticography study in macaques by Chao and colleagues (*24*). The study showed that prediction errors evoked in tone sequences trigger feedback signals from prefrontal to anterior temporal and early auditory cortex in alpha and beta frequency bands. Extending these previous results, our findings suggest that surprisal initiates bottom-up prediction errors, indicative of imprecise predictions, and top-down updates to predictions at processing stages of shorter events to facilitate perception of new words.

As an interim conclusion, our findings have two important implications for frameworks of prediction and prediction error: First, we show that a multilayered hierarchy of predictive coding [e.g., (*18*)] applies well to temporoparietal language processing. Second, predictive coding remains a viable account of neural processing, also when put to test using complex temporal dependencies underlying real-life stimuli.

### Implications for a larger network perspective on the event-based prediction hierarchy

Dual stream models of language propose that speech processing is organized along a ventral and a dorsal stream (*64*). In the present study, we found a hierarchy of speech prediction along the dorsal stream, which emanated from early auditory cortex and extended well into parietal cortex (Fig. 4B).

This result may seem at odds with other studies showing an additional mirror-symmetric ventral gradient, in which more complex speech features are represented in more anterior temporal regions (*65*). The ventral stream has been proposed to chunk speech features into increasingly abstract concepts irrespective of their temporal presentation order (*66*). In contrast, here, we modeled context representations by respecting the temporal order of words, that is, the HM-LSTM integrates incoming words into an event until a change in situational features of words indicates the end of an event and a new event is created. Hence, the ventral stream may contribute to hierarchical speech prediction by exploiting another, more nested facet of context.

The inferior frontal gyrus (IFG), alongside premotor cortex, is deemed the apex of the dorsal stream (*64*), yet, here, we considered only the role of temporoparietal cortex in speech prediction. Previous studies showed that activity in IFG relies on longer time scales of speech being intact (*27*, *67*), that connectivity between IFG and superior temporal gyrus is driven by expectations (*68*, *69*), and that right IFG is sensitive to the violation of nonlocal regularities (*70*, *71*). While this suggests an interplay between frontal and temporoparietal regions in hierarchical speech prediction, the precise anticipatory mechanisms that IFG exerts cognitive control over are just as unclear as how top-down cognitive control and bottom-up sensory input are balanced along the hierarchy.

Another open question is the cross-linguistic generalizability of the observed speech prediction hierarchy. The present study focused on German, which is—like to any other language—characterized by its unique linguistic structure shaping also neural computations. For example, alphabetic languages such as German versus tonal languages such as Chinese engage different neural systems for phonological processing (*72*). On the other hand, higher-level linguistic processing in temporal and parietal cortex has been shown to be consistent across a diverse set of languages and language families (*73*). As the event structure of speech relies on these higher-level semantic representations, this opens up the possibility that event-based predictive processing along the temporoparietal pathway is a universal function of language comprehension.

Beyond short-term semantic context, long-term knowledge also facilitates speech prediction. In theory, both memory systems can be couched into the larger framework of the dual reference frame system (*74*), where flexible sensory knowledge in parietal cortex interacts with stable conceptual knowledge in hippocampus. Consistent with the key characteristics of the speech prediction hierarchy, hippocampus codes for boundaries in the environment (*75*), hierarchically organizes memories (*76*), and engages in predictive coding (*77*). As parietal cortex has been shown to interface with hippocampus at event boundaries of longer time scales during movie watching (*41*), we speculate that the hierarchy of speech prediction might extend from receptive windows in parietal cortex to hippocampus.

The event-based prediction hierarchy relies on a set of neural computations—i.e., event segmentation, temporal receptive windows, and predictive coding—available beyond the domain of language. For example, taking a walk through the city and then taking a break in the garden constitute two events that are distinguishable not only by the change of location when presented as a narrated story but also by the change of visual (sight of traffic lights versus trees) or olfactory features (smell of car exhausts versus flowers) when experienced in a natural environment. Our results thereby encourage future studies to probe the generalizability of the event-based prediction hierarchy to other species, sensory modalities, and cognitive functions.

The present study bridges the gap between the hierarchical, temporally structured organization of context in language comprehension on the one hand and the more general principles of hierarchical predictive processing in the cerebral cortex on the other hand. Combining continuously narrated speech, artificial neural networks, and fMRI building on these networks’ output allowed us, first, to sample the natural dynamic range of word-to-word changes in predictiveness over a multilevel hierarchy. Second, we were able to systematically compare the neural effects of different contextual updating mechanisms.

Our data demonstrate that the prediction processes in language comprehension build on an event-based organization of semantic context along the temporoparietal pathway. Not least, we posit that such an event-based organization provides a blueprint for a contextually diverse yet computationally efficient network architecture of anticipatory processing in complex naturalistic environments.

## MATERIALS AND METHODS

### Participants

Thirty-seven healthy, young students took part in the fMRI listening study. The final sample included *N* = 34 participants (18 to 32 years; *M* = 24.65; 18 females), as data from 1 participant were excluded from all analyses because of strong head movement throughout the recording [mean framewise displacement, >2 SD above group average (*78*)], and two experimental sessions were aborted because participants reported to not understand speech against noise. Another 26 students (19 to 32 years; *M* = 23.54; 17 females) took part in the behavioral self-paced reading study.

All participants were right-handed German native speakers who reported no neurological, psychiatric, or hearing disorders. Participants gave written informed consent and received an expense allowance of €10/hour of testing. The study was conducted in accordance with the Declaration of Helsinki and was approved by the local ethics committee of the University of Lübeck.

### Stimulus materials

As a speech stimulus in the fMRI listening study, we used the first 64 min of an audio recording featuring H. Müller, a Nobel laureate in literature, reminiscing about her childhood as part of the German-speaking minority in the Romanian Banat (“Die Nacht ist aus Tinte gemacht,” 2009). To emulate an acoustically challenging scenario in which listeners are likely to make use of the predictability of speech (*79*), this recording was energetically masked by a stream of concatenated 5-s sound textures at a signal-to-noise ratio of 0 dB. Sound textures were synthesized from the spectrotemporal modulation content of 192 natural sounds [i.e., human and animal vocalizations, music, tools, and nature scenes (*80*)], so that the noise stream did not provide any semantic content potentially interfering with the prediction of upcoming speech. The order in which sound textures were arranged was randomized across participants. For more details on how sound textures in the present experiment were generated and how they were processed in auditory cortex, see (*48*).

The monaural speech and noise streams were sampled to 44.1 kHz, and custom filters specific to the left and right channels of the earphones used in the fMRI experiment were applied for frequency response equalization. Last, speech-in-noise stimuli were divided into eight excerpts of 8 min, which served as independent runs in the experiment.

A trained human external agent literally transcribed the speech stream. The text transcript comprised 9446 words, which were used as stimuli in the self-paced reading task and as input to our language models. To automatically determine the onset and offset times of all spoken words and phonemes, we used the web service of the Bavarian Archive for Speech Signals (*81*): First, the text transcript was transformed to a canonical phonetic transcript encoded in SAM-PA by the G2P module. Second, the most likely pronunciation for the phonetic transcript was determined by a Markov model and aligned to the speech recording by the MAUS module. Fourteen part-of-speech tags were assigned to the words in the text transcript using the pretrained German language model de_core_news_sm (2.2.5) from spaCy (https://spacy.io/). On the basis of these tags, words were classified as content or function words. Word frequencies were derived from the subtitle-based SUBTLEX-DE corpus (*82*) and transformed to standardized Zipf values (*83*) operating on a logarithmic scale from about 1 (word with a frequency of 1 per 100 million words) to 7 (1 per 1000 words). The Zipf value of a word not observed in the corpus was 1.59 (i.e., the smallest possible value).

### Experimental procedures

#### Behavioral self-paced reading task

While the transcribed story was presented word by word on a noncumulative display, participants had the task to read each word once at a comfortable pace and quickly press a button to reveal the next word as soon as they had finished reading. A timeout of 6 s was implemented. The time interval between word appearance and button press was logged as the reading time. After each run, participants answered three four-option multiple-choice questions on the plot of the story (performance: *Ra* = 58.33 to 100% correct, *M* = 79.17%, and SD = 10.87%) and took a self-paced break. In total, each participant completed four of eight runs, which were randomly selected and presented in chronological order. Throughout the reading task, we recorded movement and pupil dilation of the participants’ left eye at a sampling rate of 250 Hz in one continuous shot with an eye tracker (EyeLink 1000, SR Research). Eye tracking data were not analyzed in the present study.

The experiment was controlled via the Psychophysics Toolbox (*84*) in MATLAB (R2017b, MathWorks). All words were presented 20% off from the left edge of the screen in white Arial font on a gray background with a visual angle of approximately 18°. Participants used a response pad (URP48, The Black Box ToolKit) to navigate the experiment with their right index finger. The experimental session took approximately 40 min.

#### fMRI listening task

We instructed participants to carefully listen to the story while ignoring the competing stream of sound textures and the MRI scanner noise in the background. Each of the eight runs was initialized by 10 baseline MRI volumes, after which a white fixation cross appeared in the middle of a gray screen and playback of the 8-min audio recording started. MRI recording stopped with the end of playback, and participants successively answered the same questions used in the self-paced reading task via a response pad with four buttons (HHSC-2x4-C, Current Designs). On average, participants answered 65.5% of the questions correctly (*Ra* = 38 to 100% and SD = 15.9%). There was a 20-s break between consecutive runs.

The experiment was run in MATLAB (R2016b) using the Psychophysics Toolbox. Stimuli were presented at a subjectively comfortable sound pressure level via insert earphones (S14, SENSIMETRICS) covered with circumaural air cushions. The experimenters monitored whether participants kept their eyes open throughout the experiment via an eye tracker.

#### MRI data acquisition

MRI data were collected on a 3-T Siemens MAGNETOM Skyra scanner using a 64-channel head coil. During the listening task, continuous whole-brain fMRI data were acquired in eight separate runs using an echo-planar imaging sequence [repetition time (TR) = 947 ms, echo time (TE) = 28 ms, flip angle = 60°, voxel size = 2.5 mm by 2.5 mm by 2.5 mm, slice thickness = 2.5 mm, matrix size = 80 by 80, field of view = 200 mm by 200 mm, and simultaneous multislice factor = 4]. Fifty-two axial slices were scanned in interleaved order. For each run, 519 volumes were recorded.

Before each second run, field maps were acquired with a gradient echo sequence (TR = 610 ms, TE_1_ = 4.92 ms, TE_2_ = 7.38 ms, flip angle = 60°, voxel size = 2.5 mm by 2.5 mm by 2.75 mm, matrix size = 80 by 80, axial slice number = 62, slice thickness = 2.5 mm, and slice gap = 10%). In the end of an experimental session, anatomical images were acquired using a T1-weighted magnetization-prepared rapid gradient-echo (MP-RAGE) sequence (TR = 2400 ms, TE = 3.16 ms, flip angle = 8°, voxel size = 1 mm by 1 mm by 1 mm, matrix size = 256 by 256, and sagittal slice number = 176) and a T2-weighted sampling perfection with application-optimized contrasts using different flip angle evolution (SPACE) sequence (TR = 3200 ms, TE = 449 ms, flip angle = 120°, voxel size = 1 mm by 1 mm by 1 mm, matrix size = 256 by 256, and sagittal slice number = 176).

### Modeling the predictiveness of context at multiple time scales

We trained two versions of LSTM networks with five layers to predict the next word in the story given a sequence of semantic context: a continuously updating LSTM where information is fed to a higher layer with each upcoming word and a competing sparsely updating HM-LSTM where information is fed to a higher layer only at the end of an event. The predictiveness of context at multiple time scales was read out from single layers of both language models for each word in the story presented to participants in experiments. Ultimately, we tested how closely these derivatives of different network architectures match the signatures of behavioral and neural prediction processes.

#### Representing words in vector space

In natural language processing, it is common to represent a word by its linguistic features in the form of high-dimensional vectors (or embeddings). As the German language is morphologically rich and flexibly combines words into new compounds, there are many rare words for which language models cannot learn good (if any) vector representations on the word level. Therefore, we mapped all texts used for training, validating, and testing our language models to pretrained subword vectors publicly available in the BPEmb collection (*85*). These embeddings allow for the representation of any word by a combination of 100-dimensional subwords from a finite vocabulary of 100,000 subwords. We further reduced this vocabulary to those subwords that appeared at least once in any of our texts (i.e., number of subwords in vocabulary *v* = 91,645). See text S1 for a detailed description of the BPEmb vocabulary.

Matching our texts to subwords and their respective embeddings in the BPEmb vocabulary yielded the embedded text *t* ∈ *R*^*w*×*e*^, where *w* is the number of words and *e* = 100 is the number of vector dimensions. On average, a word in the story was represented by 1.07 subwords (*Ra* = 1 to 6 and SD = 0.33). As single words were encoded by only one subword in 94.25% of cases, we will refer to subwords as words from here on.

#### Architecture of language models

When listening to a story, a fused representation of all spoken words {*w*_1_, *w*_2_, …, *w_p_*} is maintained in memory and used as context information to make a prediction about the upcoming word *w*_*p*+1_. In natural language processing, this memory formation is implemented via recurrent connections between the states of adjacent neural network cells. The hidden state **h**_*p*−1_ stores all relevant context and is sequentially passed to the next cell where it is updated with information from word **w***_p_*.

As such a simple recurrent neural network tends to memorize only the most recent past, the more complex LSTM (*45*) became a standard model in time series forecasting. In an LSTM cell, the state is split in two vectors: The cell state **c***_p_* acts as long-term memory, whereas the hidden state **h***_p_* incorporates information relevant to the cell output (i.e., the prediction of the next word). The integration of new information and the information flow between the two memory systems are controlled by three gating mechanisms.

When stacking multiple LSTM cells on top of each other, semantic context gets hierarchically organized in the model, with lower layers coding for short-term dependencies and higher layers coding for long-term dependencies between words. The bottom-up input to the first layer remains to be the embedded word **w***_p_*. However, the lower layer’s hidden state $h_{p}^{l-1}$ becomes the input to a cell from the second layer on. The hidden state and cell state are updated at each layer with every new bottom-up input to the model.

A competing model that has been shown to slightly outperform the continuously updating (vanilla) LSTM in character-level language modeling is the HM-LSTM (*42*). This sparsely updating HM-LSTM uses a revised updating rule where information from the lower layer is only fed forward at the end of an event. The end of an event is marked by a boundary detector when situational features change. Here, we used the simplified version of the HM-LSTM (*86*) with no top-down connections. See text S2 for a detailed description of the model architecture including all relevant formulas.

#### Prediction of the next word

LSTM and HM-LSTM cells form the representations of information relevant to speech prediction, whereas the actual prediction of the next word takes place in the output module. Here, hidden states at word position *p* are combined across the different layers of the language model. The combined hidden state $h_{p}^{r}$ is mapped to a fully connected dense layer of as many neurons as there are words in the vocabulary and squashed to values in the interval [0,1], which sum to 1 (i.e., softmax function). Each neuron in resulting vector **d***_p_* indexes one particular word in vocabulary *v* and denotes its probability of being the next word. Last, the word referring to the highest probability in the distribution is chosen as the predicted next word ***s****_p_* in a story. See text S3 for a detailed description of word prediction including all relevant formulas.

#### Training and evaluation of language models

The objective of our language models was to minimize the difference between the “predicted” probability distribution **d***_p_* (i.e., a vector of probabilities ranging from 0 to 1) and the “actual” probability distribution corresponding to the next word in a text (i.e., a vector of zeros with a one-hot encoded target word). To this end, we trained models on mini batches of 16 independent text sequences with 500 words each and monitored model performance by means of categorical cross-entropy between the predicted and actual probability distribution of each word in a sequence. On the basis of model performance, trainable parameters were updated after each mini batch using the Adam algorithm for stochastic gradient optimization (*87*).

Our text corpus comprised more than 130 million words including 4400 political speeches (*88*) and 1200 fictional and popular scientific books. All texts had at least 500 words; metadata, page numbers, references, and punctuations (except for hyphenated compound words) were removed from documents. A held-out set of 10 randomly selected documents was used for validation after each epoch of training (i.e., going through the complete training set once) and allowed us to detect overfitting on the training set. Training automatically stopped after model performance did not increase over two epochs for the validation dataset.

Using a context window of 500 words, we aimed at roughly modeling time scales of the length of common linguistic units in written language (i.e., words, phrases, sentences, and paragraphs). Therefore, we only used a small range of values from three to seven to find the number of layers—intended to represent distinct time scales—best suited to make good predictions. In addition, we tuned the number of units in LSTM and HM-LSTM cells of language models, using values from 50 to 500 in steps of 50. Hyperparameters were evaluated on a single epoch using grid search, and the best combination of hyperparameters was chosen on the basis of the performance on the validation set. Our final language models had five LSTM or HM-LSTM layers with 300 units each and an output module. The LSTM model had 31,428,745 and the HM-LSTM model had 31,431,570 trainable parameters. Models were trained and evaluated with custom scripts in TensorFlow 2.1. See text S4 for a detailed description of architectural choices.

#### Deriving the predictiveness of time scales by “masking” language models

We used each trained language model to determine the predictiveness of semantic context in the story presented to participants in the behavioral and fMRI experiment. First, predictiveness was read out from full models: We iteratively selected each word in the story as a target word and fed all 500 context words preceding the target word to our language models. Note that the context for target words in the very beginning of the story comprised less than 500 words. The predicted probability of each word in the vocabulary was extracted from distribution *d_p_* in the output module.

Second, predictiveness was read out from masked models, where we allowed information to freely flow through networks yet only considered semantic context represented at single layers to generate the predicted probability distribution. These time scale–resolved probabilities were created by setting the weight matrix $W_{r}^{l}$ of pretrained models to zero for all layers of no interest, so that the hidden state of only one layer is passed to the softmax function and all other layers have no bearing on the final prediction. We iteratively set all but one layer to zero, with each layer being the only one influencing predictions once, resulting in five masked outputs for each language model.

We derived three measures of predictiveness from probability distributions. Our primary measure was the degree of surprisal associated with the occurrence of a word given its context. Word surprisal is the negative logarithm of the probability assigned to the actual next word in a story.

Secondary measures of predictiveness were used to explore the specificity of the processing hierarchy to only some aspects of the prediction processes. Entropy reflects the amount of uncertainty across the whole probability distribution of candidate words, which is the negative sum of probabilities multiplied by their natural logarithm. When high probabilities are assigned to only one or few words in the vocabulary, entropy is low. On the other hand, entropy is high when semantic context is not informative enough to narrow predictions down to a limited set of words, resulting in similar probabilities for all candidate words. As all information necessary to determine the entropy of a word is already available to participants before word presentation, entropy of word *w_p_* was ascribed to the previous word *w*_*p*−1_. Whereas entropy quantifies the overall difficulty of making any definite prediction, surprisal quantifies the availability of information about the actual next word.

Another secondary measure of predictiveness was the relatedness of the predicted next word to the actual next word. This word dissimilarity is expressed as 1 minus the correlation of respective word embeddings. A high positive product-moment correlation indicates that the prediction is linguistically close to the target word, although the model prediction might have been incorrect.

All three measures were calculated for each word in the story, separately for full models and five masked models. This yielded an 18-dimensional feature space of predictiveness for the LSTM and the HM-LSTM model, which was linked to BOLD activity and reading times in our analysis.

In addition, we created a metric to dissociate the neural effects of predictiveness from more low-level effects of semantic incongruence between target words and their preceding context. To this end, we correlated the embedding of each function word in the story with the average embedding of a context window and subtracted the resulting product-moment correlation coefficients from 1 (*89*). This measure of contextual incongruence was calculated at five time scales corresponding to a logarithmic increase in context length (i.e., 2, 4, 8, 16, and 32 words).

To determine the temporal integration windows of layers, we scrambled input to the language models at nine levels of granularity corresponding to a binary logarithmic increase in the length of intact context (i.e., context windows of 1 to 256 words). For each layer, we fit linear functions to word surprisal across context windows and extracted slope parameters indicating how much a layer benefits from longer context being available when predicting the next word. On the second level, we fit linear functions to these layer-specific integration windows to determine the context benefit of higher layers over shorter layers. Resulting model-specific slopes were compared to a null distribution of slopes computed by shuffling the integration windows across layers (*n* = 10,000). In addition, slopes were compared between language models by means of a Monte Carlo approximated permutation test (*n* = 10,000) on the difference of means.

#### Convolving features with the HRF

We used three classes of features to model brain responses: 18 features of predictiveness (i.e., surprisal, entropy, and dissimilarity for the full model and for the five masked models), 3 linguistic features, and 9 acoustic features. While we were primarily interested in modeling the effects of predictiveness, linguistic and acoustic features were used as nuisance regressors potentially covarying with predictiveness. Linguistic features included information about when words were presented (coded as 1), whether they were content or function words (coded as 1 and −1), and which frequency they had. In (*48*), we decomposed the speech-in-noise stimuli into a 288-dimensional acoustic space of spectral, temporal, and spectrotemporal modulations, which was derived from a filter bank modeling auditory processing (*90*). Here, we reduced the number of acoustic features to the first nine principal components, which explained more than 80% of variance in the original acoustic space. All features were *z*-scored per run.

A set of 500 scrambled features of predictiveness was generated, which was used to estimate null distributions of predictive processing. We applied the fast Fourier transform to single features, randomly shifted the phase of frequency components, and inverted the transform to project the data back into the time domain. This preserved the power spectra of features but disrupted the temporal alignment of frequencies. See text S5 for a detailed description of convolving features with the HRF.

### Data analysis

See text S6 for a detailed description of structural and functional MRI data preprocessing.

#### Selection of ROIs

We hypothesized that the speech prediction hierarchy is represented as a gradient along a temporoparietal pathway. This rather coarse ROI was further refined to only include regions implicated in speech processing. To this end, we used intersubject correlation (*49*) as a measure of neural activity consistently evoked across participants listening to speech in noise. As we were primarily interested in shared responses to the speech stream, this approach allowed us to leverage the inconsistency of the noise stream across participants. The presentation of sound textures in different order likely evoked more heterogeneous neural responses, leading to a diminished shared representation of the noise stream. Therefore, we inferred that the shared neural responses we observed were largely driven by the speech stream, which was the same for all participants.

At the first level, hyperaligned functional time series of each participant (see text S6) were concatenated across experimental runs and correlated with every other participant on a vertex-by-vertex basis, resulting in pairwise maps of intersubject product-moment correlations. A group map was created by calculating the median correlation coefficient across pairs of participants for each vertex. At the second level, median correlation coefficients were chosen as a nonparametric test statistic to account for inflated false-positive rates, and a bootstrap hypothesis test with 10,000 iterations was applied. To create the null distribution, we iteratively resampled participants with replacement and derived median group maps from their pairwise correlation maps. When the same participant was sampled more than once in a bootstrap iteration, the pairwise correlation map of that participant with herself was not included in the computation of the group map. The actual median intersubject correlation was ranked against the normalized null distribution to obtain a *P* value for each vertex. Intersubject correlations were computed with the Python package BrainIAK (*91*) following the recommendations of Nastase and colleagues (*49*).

Last, we used a multimodal parcellation (*51*) to select those lateral temporal and parietal parcels of which at least 80% of the vertices had a significant intersubject correlation in one hemisphere [*P* < 0.01, adjusted for false discovery rate (FDR) (*92*)]. The following parcels were included in the ROI: early auditory cortex, auditory association cortex, lateral temporal cortex, temporo-parieto-occipital junction, inferior parietal cortex, and superior parietal cortex. As the temporal middle temporal (MT)+ complex is thought to be mainly involved in visual processing, this region was not considered an appropriate candidate parcel. All further analyses including MRI data were limited to the temporoparietal ROI, which was organized along the anterior-posterior (left, 124 mm; right, 167 mm) and inferior-superior axis (left, 234 mm; right, 212 mm).

#### Functional data analysis

The starting point of our analyses was the question whether the time scales of speech prediction organize along a temporoparietal processing hierarchy. In a forward model, we encoded the predictiveness of time scales into univariate neural activity and fit a gradient along the peak locations sensitive to surprisal at specific time scales. Next, we compared the explanatory power of both language models in a supplementary backward model, which decoded surprisal at different time scales from multivariate patterns of neural activity in temporoparietal parcels. Last, we modeled functional connectivity between peak locations to test whether the time scales of surprisal gate the information flow along the gradient.

##### Encoding model

The encoding approach [similar to, e.g., (*93*)] allowed us to quantify for each temporoparietal vertex, which features of predictiveness it preferentially represents. Two separate encoding models were estimated for each vertex in the ROI of single participants, one for each language model. Besides the features of predictiveness specific to language models, both models included the same linguistic and acoustic features as nuisance regressors. We modeled neural activity as a function of the HRF-convolved features characterizing speech and noise stimuli by

a=Sw+ϵ

where **a**^samples × 1^ is the activity vector (or BOLD time course) corresponding to a vertex, *S*^samples × features^ is the stimulus matrix of features, **w**^features × 1^ is a vector of estimated model weights, and **ϵ**^samples × 1^ is a vector of random noise.

All models were estimated using ridge regression with fourfold cross-validation. We paired odd-numbered functional runs with their subsequent even-numbered run, resulting in four data splits per participant. Each of the four data splits was selected as a testing set once; all other data splits were used as a training set. Within each fold, generalized cross-validation (*94*) was carried out on the training set to find an optimal estimate of regularization parameter λ from the data, searching 100 values evenly spaced on a logarithmic scale from 10^−5^ to 10^8^. Weights of predictiveness were extracted from the model fit with the optimal regularization parameter and averaged across cross-validation folds to obtain stable weights.

To evaluate the performance of encoding models and their ability to generalize to new data, we applied the weights estimated on the training set to the features of the held-out testing set in each cross-validation fold. The predicted BOLD time series was correlated with the actual BOLD time series. The resulting product-moment correlation coefficient is the encoding accuracy, which was averaged across cross-validation folds and Fisher *z*-transformed.

In addition, we created null distributions of weights and encoding accuracies by estimating forward models on the scrambled features of predictiveness [similar to, e.g., (*95*)]. We set up 500 separate models, which included scrambled features of predictiveness but intact linguistic and acoustic features. Models were estimated largely following the cross-validation scheme outlined for observed data. However, we reused the optimal regularization parameters from nonscrambled models of corresponding folds. All ridge regression models were implemented using the RidgeCV function in the Python package scikit-learn (*96*).

##### Peak selection

For both language models, we derived five temporoparietal maps in the left and right hemispheres of single participants: one weight map for each time scale of word surprisal. Maps represented the sensitivity of brain regions to time scale surprisal; positive weights indicate increasing BOLD activity to more surprising words.

To illustrate the location and extent of brain regions modulated by time scale surprisal, we performed an analysis similar to cluster-based permutation tests in Fieldtrip (*97*). For each time scale, vertex-wise weights observed across participants were tested against zero by means of a one-sample *t* test. We combined a vertex into a cluster with its adjacent vertices if it was significant at an alpha level of 0.05 and had at least two significant neighbors. We clustered vertices with negative *t* values separately from vertices with positive *t* values. The summed *t* value of an observed cluster served as the cluster-level statistic and was compared with a Monte Carlo approximated null distribution of summed *t* values. This null distribution was created by performing clustering on the scrambled partitions of time scale–specific weight maps and selecting the largest summed *t* value for each partition. An observed cluster was considered significant if its summed *t* value was exceeded by no more than 2.5% of the summed *t* values from scrambled partitions.

Beyond this rather coarse mapping of temporoparietal brain regions onto the time scales of surprisal, our main analysis focused on how time scale–specific peak locations distribute along the inferior-superior axis only. Notably, we hypothesized that a hierarchy of speech prediction evolves from temporal to parietal areas, which correspond to the inferior-superior axis of our ROI. A window with a height of 2 mm was shifted along the inferior-superior axis of the temporoparietal ROI in steps of 1 mm. All weights of a time scale falling into the window were averaged, thereby collapsing across the anterior-posterior axis. The resulting one-dimensional weight profile of time scale surprisal spanned inferior to superior locations and was smoothed using robust linear regression over a window of 70 mm. For each unilateral weight profile of single participants, local maxima (i.e., a sample larger than its two neighboring samples) were determined.

We applied two different approaches to selecting one peak location for each time scale from these local maxima. In the unconstrained approach of peak selection, the local maximum with the highest positive value was defined as a peak. As this approach makes it hard to find a consistent order of time scales when surprisal is processed not only along the dorsal but also along the ventral processing stream, we also applied a constrained peak selection approach explicitly targeting the dorsal stream. Here, the peak of surprisal at the first time scale had to be in the inferior half of the axis (i.e., temporal regions), and peaks of surprisal at longer time scales had to be superior to the peak of the first time scale. Whenever no time scale peak could be defined, the largest positive value was selected. Both peak selection approaches yielded five time scale–specific coordinates on the inferior-superior axis for each participant, hemisphere, and language model.

##### Gradient fitting

We fit linear functions to coordinates of single participants across the time scales of surprisal. Models included an intercept term, and the slope parameter was extracted from each fit. A positive slope indicates a gradient of time scale surprisal, where surprisal at shorter time scales is represented in more (inferior) temporal regions than longer time scales, which are represented in more (superior) parietal regions. We tested grand-average slope parameters against a null distribution of slopes with 10,000 partitions, which was created by randomly shuffling the coordinates of single participants across the time scales of surprisal and recalculating their slopes. As the first time scale was preset to have the most inferior coordinate in the constrained peak selection approach, this specific coordinate was not shuffled when calculating the null distribution for this approach. To compare slope parameters between language models, we performed a Monte Carlo approximated permutation test with 10,000 iterations, using the difference of means as a test statistic.

As secondary analyses, gradients of predictive processing were also calculated for the time scales of word entropy and dissimilarity. In a control analysis, a gradient was fit to time scale peaks following the same procedure described above but replacing the features of predictiveness by contextual incongruence when estimating forward models.

In addition, we applied unconstrained peak selection to weight maps, which were rotated around the full circle in steps of 5° before collapsing across the first dimension. After deriving test statistics for slope parameters at each rotation as described for nonrotated slope parameters, cluster-based permutation tests were calculated following a similar procedure as described for time scale–specific maps of regression weights before but, this time, performing clustering in the rotation domain.

To round off the encoding analysis, we compared temporoparietal encoding accuracies between both language models. As we were interested in effects specific to the predictiveness of speech, encoding accuracies were *z*-scored to the null distribution of accuracies from the scrambled features of predictiveness. A cluster-based permutation paired-sample *t* test was calculated (*n* = 1000; vertex-specific alpha level, 0.05; cluster-specific alpha level, 0.05). In comparison to the cluster test described above for the weight maps, here, we created a null distribution of summed *t* values by contrasting the accuracies of language models whose labels had been randomly shuffled in single participants.

##### Functional connectivity

To model the information flow between brain regions sensitive to surprisal at the different time scales, we identified five unique seeds for both language models in each temporoparietal hemisphere. On the inferior-superior axis, we reused the grand-median coordinate of each time scale as localized in the constrained peak selection approach. The corresponding coordinate on the anterior-posterior axis was localized by shifting a moving average with a window centered on the inferior-superior coordinate along the anterior-posterior axis (width, 2 mm; height, 5 mm) and determining peak locations on smoothed weight profiles of single participants. Next, we placed a sphere with a radius of 5 mm on peak coordinates from both axes and averaged the BOLD time courses of vertices falling within this sphere, yielding the time scale–specific neural activity of seeds.

We expected increased information flow between seeds of adjacent time scales when one time scale becomes uninformative for the prediction of upcoming speech. This modulatory influence of surprisal on connectivity was modeled along the lines of a PPI (*52*). In a standard PPI analysis, the neural time series of one brain region is regressed onto the pointwise product of an experimental stimulus and the neural time series of another brain region. Here, we extended this approach by creating time scale–specific interactions: BOLD time series of seeds were multiplied by their corresponding HRF-convolved surprisal time series but not any of the surprisal time series at another time scale.

Functional connectivity was calculated for both language models in each participant and hemisphere. We set up five regression models, with every seed being selected as a target once. The physiological (BOLD) time series of the target seed was mapped onto the physiological, psychological (time scale–specific surprisal), and psychophysiological time series from all other (predictor) seeds. Models were estimated within the same cross-validation scheme outlined for the encoding model. We extracted all four weights from PPI terms of each target seed and arranged weights in a five-by-five matrix, with target seeds on the main diagonal and predictor seeds off the diagonal. This matrix of observed PPIs was compared to a matrix with hypothesized interaction weights: The diagonals below and above the main diagonal were set to 1 (indicating increased coupling when surprisal at a neighboring time scale is high), and all other items were set to −1. We calculated the Euclidean distance of single-participant matrices to this hypothesized matrix. The mean of observed Euclidean distances was compared to a null distribution of 10,000 mean Euclidean distances calculated on BOLD time series of target seeds randomly shifted in time by the number of samples in one to seven functional runs. Euclidean distances were compared between language models in each hemisphere by means of a Monte Carlo approximated permutation test (*n* = 10,000) on the difference of means.

#### Behavioral data analysis

Reading times were used to test the behavioral relevance of the predictiveness determined by our language models. Trials with reading times shorter than 0.001 s or longer than 6 s were considered invalid and excluded. Furthermore, we inverted reading times into speed and excluded trials exceeding 3 SD within a run and participant from all further analyses. On average, 1.31% of trials (*Ra* = 0.32 to 6.15% and SD = 1.12%) were removed. Participants performed the task at a mean response speed of 4.96 s^−1^ (*Ra* = 1.71 to 10.13 s^−1^ and SD = 2.08 s^−1^), with speed increasing over the four experimental runs (*P* < 0.001, *d* = 1.06; compared to a null distribution of slopes drawn from linear functions fit to the shuffled mean speed of runs). Last, reading speed was *z*-scored within runs.

For each participant, we predicted reading speed in a forward model, adopting the same cross-validated ridge regression scheme used for the analysis of fMRI data. Our feature space included the predictiveness of words and a set of nuisance regressors, namely, word frequency, word length (number of letters), content versus function words, and trial number. To account for spill-over effects from previous words on reading times (*98*), we added time-lagged versions of features to the model. As previous studies controlled for the spill-over effect on up to three words following (*99*), we shifted features by −2 to 5 word positions to illustrate the effect also returning to zero. There were no lagged versions of the predictor coding for trial number added to the model.

To investigate whether predictiveness had an effect on reading speed beyond the effect of nuisance regressors, we compared the predictive accuracy of forward models in single participants to a null distribution of accuracies from models with scrambled features of predictiveness. The performance of language models was compared by *z*-scoring the observed encoding accuracies to the null distribution and running a Monte Carlo approximated permutation test (*n* = 10,000) on the difference of means. This analysis was also carried out for the time scales of contextual dissimilarity.

## Supplementary Materials

### This PDF file includes:

Supplementary Texts S1 to S7 Figs. S1 to S6 References
