# Supplementary material for: Predicting speech from a cortical hierarchy of event-based time scales
Source: Sci Adv. 2021 Dec 3;7(49):eabi6070. doi: 10.1126/sciadv.abi6070 (PMC8641937; doi:10.1126/sciadv.abi6070)
Supplement: Supplementary file 1 — Supplementary Texts S1 to S7 Figs. S1 to S6 References [file sciadv.abi6070_sm.pdf]

Science Advances
AAAS

Supplementary Materials for

Predicting speech from a cortical hierarchy of event-based time scales

Lea-Maria Schmitt*, Julia Erb, Sarah Tune, Anna U. Rysop, Gesa Hartwigsen, Jonas Obleser*

*Corresponding author. Email: l.schmitt@uni-luebeck.de (L.-M.S.); jonas.obleser@uni-luebeck.de (J.O.)

Published 3 December 2021, *Sci. Adv.* **7**, eabi6070 (2021)
DOI: 10.1126/sciadv.abi6070

This PDF file includes:

Supplementary Texts S1 to S7
Figs. S1 to S6
References

Supplementary Text

Text S1: Mapping words to pre-trained BPEmb subword vectors

In short, the BPEmb vocabulary is based on a text corpus, which was segmented into its subwords using byte-pair encoding. That is, smaller subword units (e.g., letters) most frequently co-occurring in the corpus were iteratively merged into larger units (e.g., syllables) and added to the vocabulary until the predefined maximum of merge operations was reached (i.e., vocabulary size). The corresponding embeddings were trained with the GloVe algorithm (*101*). Importantly, the length of subwords ranged from single letters to complete words. For example, the inflected verb "[sie] fischte" ["fished"; 3rd person singular, simple past, active voice, indicative of "to fish"] consists of one subword embedding representing its word stem "fisch" and another embedding representing its suffix "te", whereas the more frequent word "Wasser" ["water"] is represented by only one embedding.

Text S2: Architecture of language models

In a simple recurrent neural network (RNN), the hidden state h_{p-1} stores all relevant context and is sequentially passed to the next cell where it is updated with information from word w_p. More specifically, the recurrent input and the bottom-up input are combined into the cell input vector g_p:

$$g_p = tanh\left(W_g w_p + U_g h_{p-1} + b_g\right),$$

where *tanh* is the activation function, $W_g \in R^{n \times e}$ and $U_g \in R^{n \times n}$ are trainable weight matrices, *n* is the number of neurons (or units), $b \in R^{n \times 1}$ is a bias term, $w_p \in R^{e \times 1}$ and $h_{p-1} \in R^{n \times 1}$.

In the continuously updating LSTM, the cell state c_p acts as long-term memory, whereas the hidden state h_p incorporates information relevant to the cell output (i.e., the prediction of the next word). The integration of new information and the information flow between the two memory systems is controlled by three gating mechanisms. First, the cell state is updated. The forget gate f_p determines which information from the previous cell state c_{p-1} has become irrelevant and should be removed by:

$$f_p = \sigma\left(W_f w_p + U_f h_{p-1} + b_f\right),$$

where σ is the sigmoid activation function. The input gate i_p determines which information from candidate state g_p should be added to the cell state by:

$$i_p = \sigma\left(W_i w_p + U_i h_{p-1} + b_i\right).$$

The new cell state c_p is created by:

$$c_p = f_p \odot c_{p-1} + i_p \odot g_p,$$

where $c_{p-1} \in R^{n\times 1}$. Second, the hidden state is updated. The output gate o_p determines which information from long-term memory c_p might become relevant shortly and should be added to the hidden state by:

$$o_p = \sigma(W_o w_p + U_o h_{p-1} + b_o).$$

The new hidden state h_p is created by:

$$h_p = o_p \odot tanh(c_p).$$

The sparsely-updating HM-LSTM employs a revised updating rule where information from the lower layer is only fed forward at the end of an event (i.e., a sequence of words closely related to each other). To this aim, z_p^l is introduced which marks the end of an event:

$$\tilde{z}_p^l = hard\ sigm(z_p^{l-p} W_z h_p^{l-1} + U_z h_{p-1}^l + b_z^l),$$

$$z_p^l = \begin{cases} 1 & \text{if } \tilde{z}_p^l > 0.5 \\ 0 & \text{otherwise,} \end{cases}$$

where *hard sigm* is the hard sigmoid activation function. If $z_p^{l-1} = 1$, the hidden state h_p^l and cell state c_p^l are computed like in a vanilla LSTM cell ("update mechanism"). Otherwise, the hidden state h_p^l and cell state c_p^l are simply the copy of h_{p-1}^l and c_{p-1}^l ("copy mechanism"), respectively.

Text S3: Prediction of the next word

LSTM and HM-LSTM cells form the representations of information relevant to speech prediction, whereas the actual prediction of the next word takes place in the output module. Here, hidden states at word position *p* are combined across the different layers of the language model by:

$$h_p^r = LReLU\left(\sum_{l=1}^{L} W_r^l h_p^l\right),$$

where *LReLU* is the leaky rectified linear unit activation function and *L* is the number of layers. The combined hidden state h_p^r is mapped to a fully connected dense layer of as many neurons as there are words in the vocabulary and squashed to values in the interval [0,1], which sum to 1:

$$d_p = softmax(W_d h_p^r + b_d),$$

where *softmax* is the squashing function, $W_d \in R^{v\times n}$ and $b_d \in R^{v\times 1}$. Each neuron in vector d_p indexes one particular word in vocabulary *v* and denotes its probability of being the next word. Finally, the word referring to the highest probability in the distribution is chosen by:

$$s_p = argmax(d_p),$$

where s_p is the predicted next word in a story.

Text S4: Training of language models

All other architectural choices were based on results from systematic ablation tests on HM-LSTM cells (*86*). Accordingly, the optimizer's initial learning rate of 0.001 was reduced by a factor of 0.02 when performance on the validation set did not improve over one epoch. Gradients were clipped at a value of 1. We applied layer normalization to all inputs after multiplication with their respective weight matrices (*102*). Further, we added an l^2-norm penalty term for weight size to the loss function ($\lambda = 0.0005$). The dense layer in the output module was excluded from normalization and regularization. No dropout of units was applied during training.

Text S5: Convolving features with the hemodynamic response function

For features of predictiveness and linguistics, we modelled (higher frequency, randomly spaced) information on the word level as hemodynamic responses sampled at the (lower frequency, equally spaced) TR of fMRI data. This was achieved by creating feature vectors of zeros corresponding to the length of a functional run with a sampling frequency of 1,000 Hz, which allowed word onsets and offsets to be represented with high temporal precision. For each word in a run, a boxcar function, which was scaled to the feature's value at that particular word, was placed on all samples falling in between word onset and offset. The resulting vector including feature values for all words in a run was convolved with SPM's canonical hemodynamic response function (HRF (*103*)) and downsampled to the TR. Acoustic features, on the other hand, were already sampled to the TR and therefore directly convolved with the HRF.

Text S6: Preprocessing structural and functional MRI

Structural MRI data preprocessing. MRI data were preprocessed with fMRIPrep 1.2.4 (*104*), which is based on Nipype 1.1.6 (*105*) and employs Nilearn 0.5.0 (*106*) in many internal operations. For each participant, the T1w image was corrected for intensity non-uniformity using N4BiasFieldCorrection (ANTs 2.1.0 (*107*)) and then skull-stripped using the OASIS template in antsBrainExtraction.sh (ANTs 2.2.0). Individual brain surfaces were reconstructed from T1w and T2w reference images using recon-all (FreeSurfer 6.0.1 (*108*)). The T1w reference image was spatially normalized to the MNI152NLin2009cAsym template (*109*) through nonlinear registration with antsRegistration (ANTs 2.2.0 (*110*)).

A brain mask was created by reconciling ANTs-derived and FreeSurfer-derived segmentations of the cortical grey matter according to a customized variation of the implementation in Mindboggle (*111*). Brain tissue segmentation of cerebrospinal fluid, white matter and grey matter was performed on the T1w reference image using FAST (FSL 5.0.9 (*112*)).

Functional MRI data preprocessing. For each functional run, BOLD time series were motion corrected using mcflirt (FSL 5.0.9 (*113*)) and slice time corrected using 3dTshift (AFNI 20160207 (*114*)). After unwarping BOLD images based on the susceptibility distortion estimated from field maps, the BOLD reference image was aligned to the native T1w reference image using boundary-based registration with six degrees of freedom (*115*) as implemented in bbregister (FreeSurfer). BOLD images were resampled to standard space. To correct for head motion, non-aggressive Automatic Removal of Motion Artifacts using Independent Component Analysis (ICA-AROMA (*116*)) was performed on the resampled and smoothed (6 mm FWHM Gaussian kernel) BOLD images. On average, 50.54 % of the maximal 200 components per functional run (*Ra* = 32.93–71.78 %, *SD* = 9.21 %) were classified as motion-related artefacts. Additional confounding noise time series like the average signal within cerebrospinal fluid and white matter as well as framewise displacement were calculated in Nipype following the definitions by Power and colleagues (*117*). A Discrete Cosine Transform (DCT) basis set of six functions with a cut-off at 0.008 Hz was generated for temporal high-pass filtering.

After running fMRIPrep, we regressed out high-pass filters as well as cerebrospinal fluid and white matter signals from the BOLD time series using 3dTproject (AFNI 19.2.24). To avoid reintroducing previously removed artefacts into the functional data (*118*), we projected the ICA-AROMA artefact components onto the additional nuisance covariates and used the residuals as predictors orthogonal to prior predictors. The denoised BOLD images were resampled to the fsaverage5 template in surface space by averaging across the cortical ribbon in 5 equally spaced steps at each vertex using trilinear interpolation. All resamplings can be performed with a single interpolation step: volumetric resamplings were performed using antsApplyTransforms (ANTs 2.1.0) with Lanczos interpolation; surface resamplings were performed using mri_vol2surf (FreeSurfer). In each functional run, the first 10 baseline volumes as well as the last volume were removed from time series and all further analysis were carried out on z-scored single-vertex BOLD time series.

Functional alignment to a common space. To account for small spatial variations in intersubject response tuning, functional time series were projected into a common space using searchlight hyperalignment across the whole cortex as described by Guntupalli and colleagues (*119*). Here, we centred a sphere (or searchlight) with a radius of 20 mm on each vertex and determined the optimal rotation of response vectors (or functional time series) within each searchlight in three iterations using Procrustes transformation. An intermediate common space was initialized by rotating one participant's response vectors to best match the responses of a randomly chosen reference participant. All other participants were successively brought into alignment, with the average of all previously rotated response vectors as a reference. In a second iteration, all original response vectors were aligned to the intermediate common space and the average of resulting rotated response vectors became the final common space. In the third iteration, hyperalignment parameters mapping single participant's original response vectors to the final common space were calculated. Parameters corresponding to vertices of

overlapping searchlights were averaged. We ran hyperalignment on four independent data splits (i.e., pairing up every fourth of eight functional runs) and averaged transformation matrices across data splits to derive final parameters for each participant. Hyperalignment was performed in PyMVPA (2.6.6 (*120*)).

Text S7: Decoding model

In our decoding approach (similar to e.g., Ref. (*93*)), we quantified how much information multiple vertices jointly contain about a feature of predictiveness. For each language model, five separate backward models were estimated in each of six temporo-parietal parcels of single participants, one for word surprisal at each timescale. We modelled timescale-specific surprisal as a function of neural activity in all vertices forming a parcel by:

$$s = Aw + \epsilon,$$

where $s^{samples \times 1}$ is the stimulus vector of a feature, $A^{samples \times vertices}$ is the activity matrix of BOLD time courses corresponding to the vertices of a parcels, $w^{vertices \times 1}$ is a vector of model weights, $\epsilon^{samples \times 1}$ is a vector of random noise.

The same cross-validation scheme as described for the encoding model was applied. However, instead of predicting BOLD activity, we here reconstructed surprisal at different timescales. By correlating the actual stimulus time series with the one predicted on the held-out testing set, we obtained the decoding accuracy of a parcel. Decoding accuracies were z-scored to the null distribution of accuracies determined for scrambled stimulus time series. We compared decoding accuracies between language models in each hemisphere, parcel and timescale by means of a Monte Carlo approximated permutation test (n = 10,000) on the difference of means. Resulting *p*-values were corrected for multiple comparisons using FDR correction.

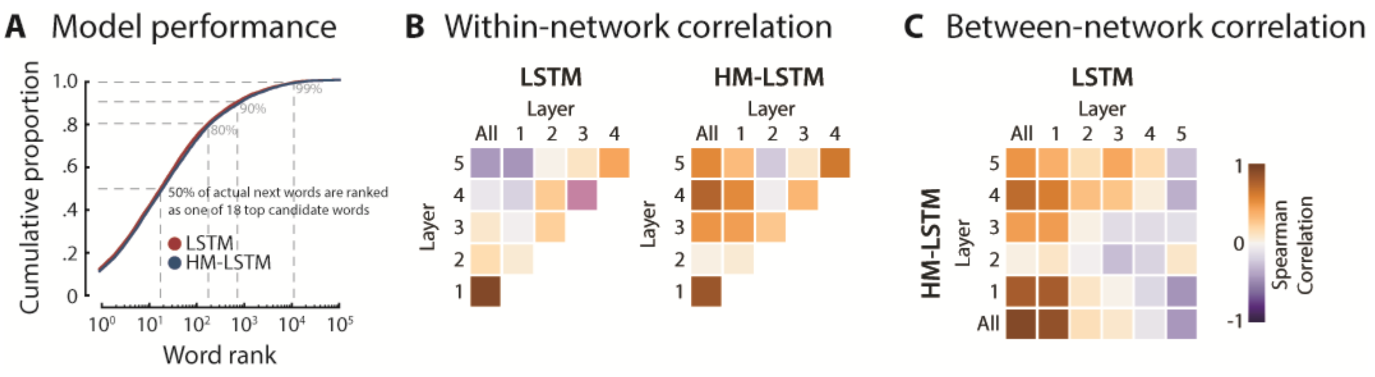

Figure S1. Evaluating language models. (A) For each language model (LSTM: red, HM-LSTM: blue), we extracted the rank of the next word from the probability distribution of all candidate words. Language models ranked more than 50 % of words in the text as one of 18 top candidates words (out of more than 90,000 words). **(B)** Spearman correlations of word surprisal between single layers of language models, separately for LSTM (left) and HM-LSTM (right). In addition, correlations with full models are shown. **(C)** Spearman correlations of word surprisal between language models.

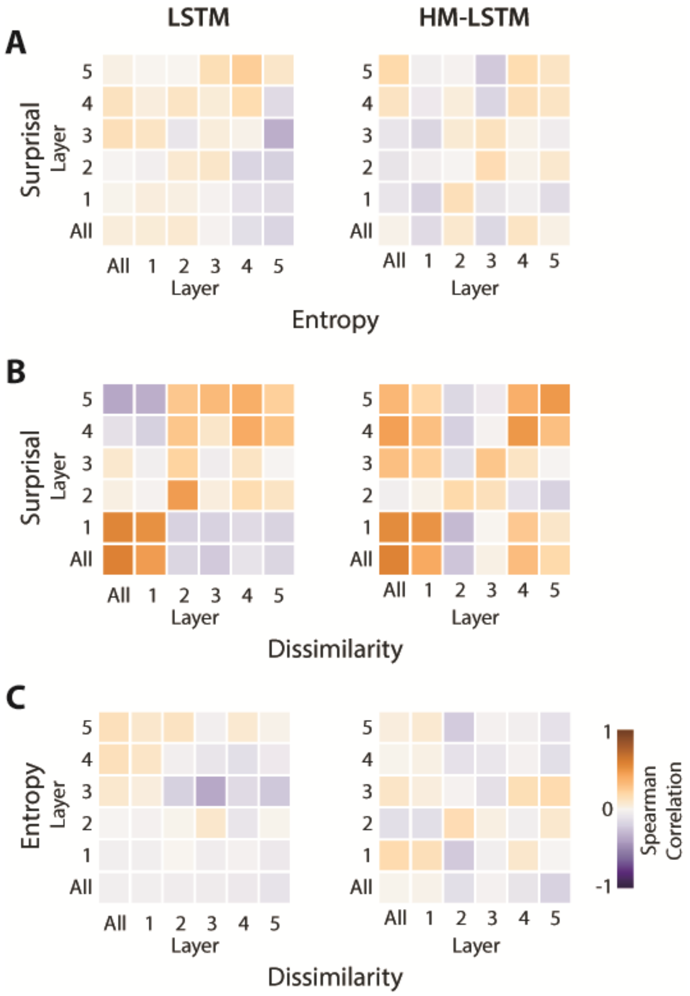

Figure S2. Inter-metric correlations. Spearman correlations between outputs from single layers of language models for **(A)** surprisal and entropy, **(B)** surprisal and dissimilarity as well as **(C)** entropy and dissimilarity, separately for LSTM (left) and HM-LSTM (right). In addition, correlations with full models are shown.

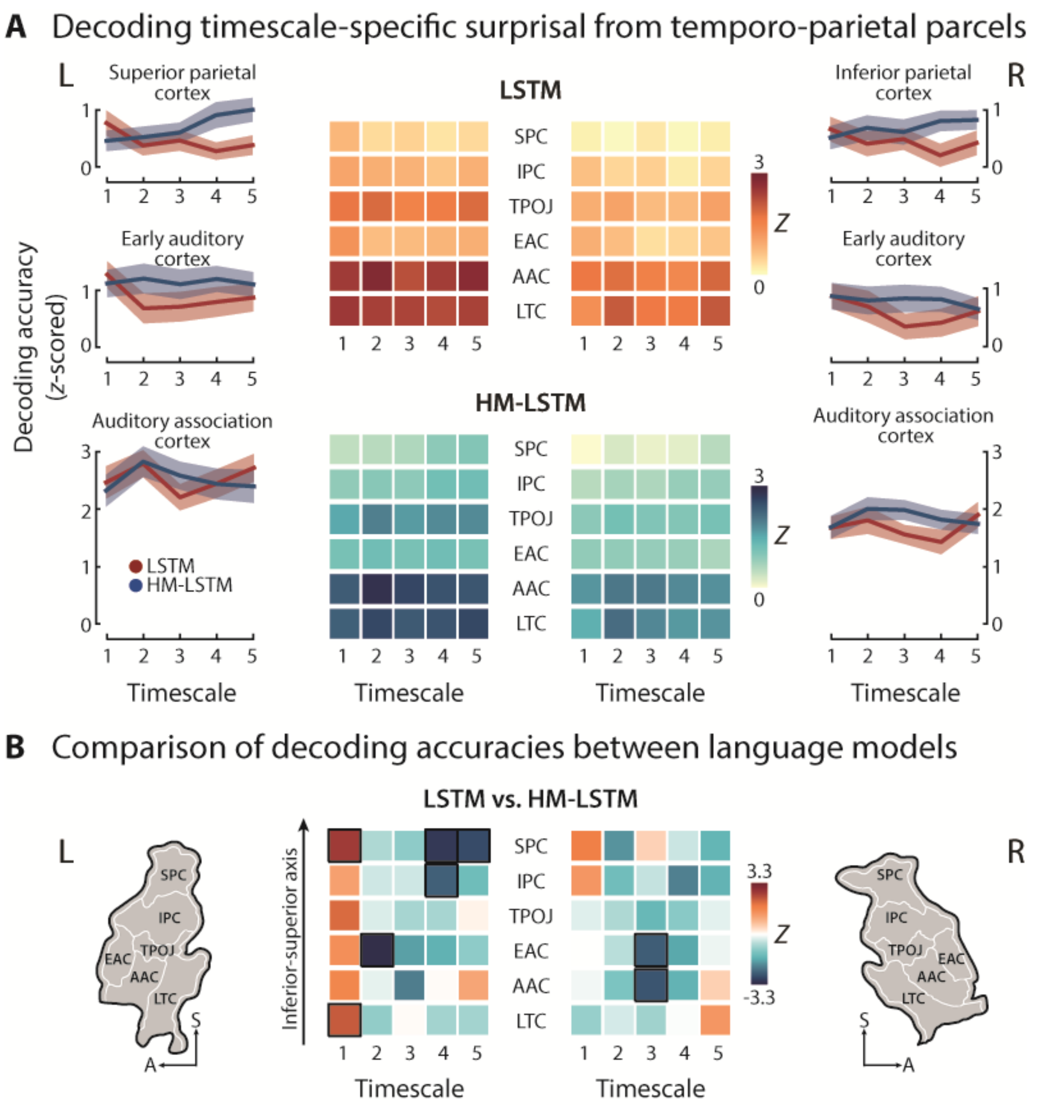

Figure S3. Decoding surprisal at multiple timescales. (A) Timescale-specific surprisal was decoded from regions of interest. Matrices depict decoding accuracies determined on held-out testing data and z-scored to null distributions drawn from scrambled surprisal, separately for the LSTM (top) and HM-LSTM (bottom) in the left (L) and right hemisphere (R). Note that comparably lower decoding accuracies in more parietal regions mirrored the lower intersubject correlations in parietal compared to temporal regions (Figure 3), which are commonly found during natural listening (e.g., Ref. (*121–123*)). This indicates an overall greater variability of neural responses in parietal regions irrespective of timescale-specific surprisal. Of note, some z-scored decoding accuracies in more superior parcels fell below an average value of 1.96. However, z-scores were indicative of significance only on the level of single participants. Line plots illustrate patterns of decoding accuracies across timescales in select regions of interest; error bands represent ±SEM. **(B)** Decoding accuracies were contrasted between language models by means of a permutation test on the mean of differences; black circles indicate $p_{FDR} < 0.05$; maps indicate location of parcels. EAC: early auditory cortex, AAC: auditory association cortex, LTC: lateral temporal cortex, TPOJ: temporo-parieto-occipital junction, IPC: inferior parietal cortex, SPC: superior parietal cortex.

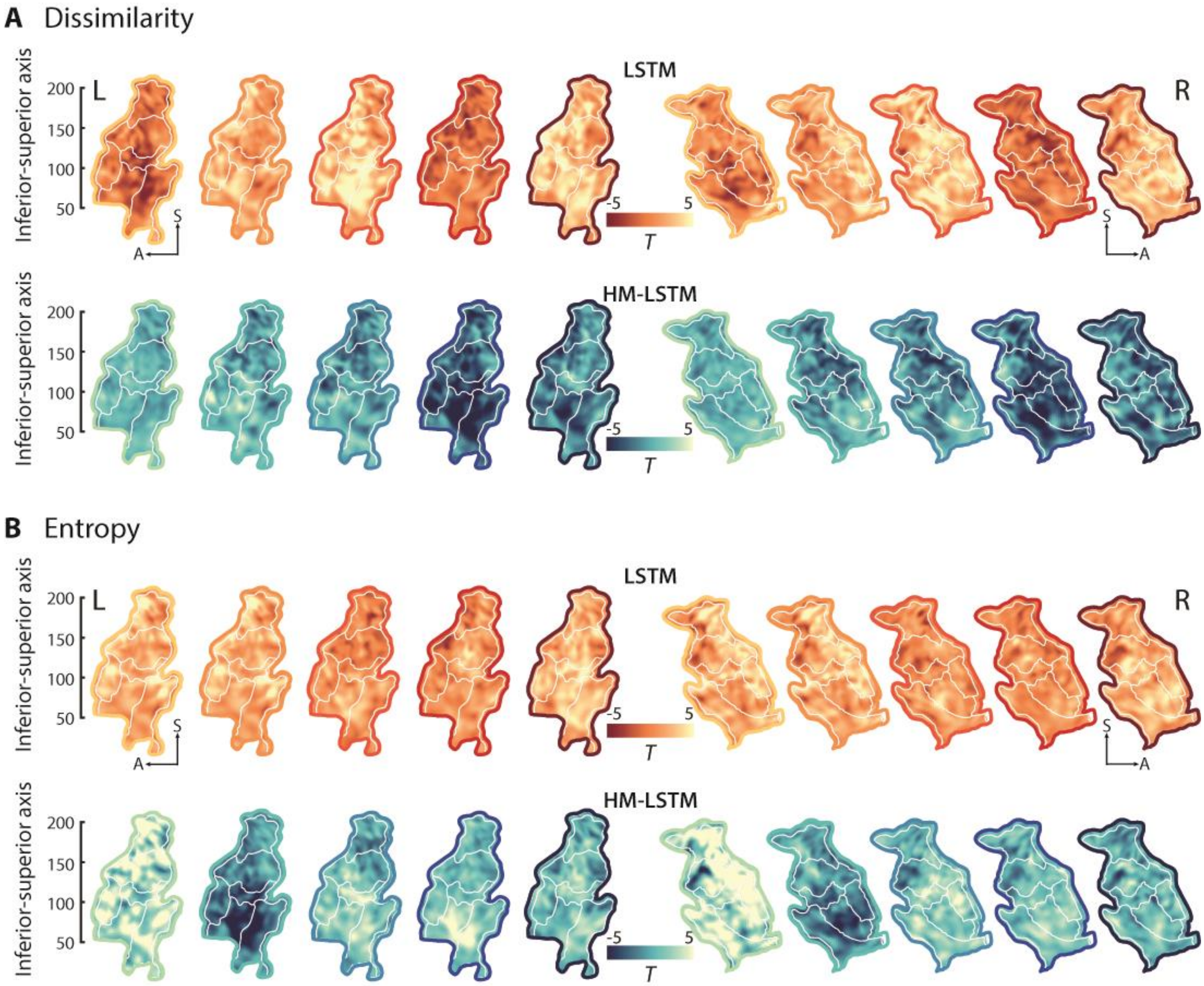

Figure S4. Encoding secondary metrics of predictiveness at multiple timescales. (A) Temporo-parietal BOLD time series were mapped onto the dissimilarity of speech derived at five timescales, separately for the continuously updating LSTM (top) and the sparsely updating HM-LSTM (bottom) in both hemispheres. Maps show *t*-values from timescale-specific weights of dissimilarity tested against zero; positive *t*-values indicate an increase of BOLD activity in response to more dissimilar words; white outlines: parcels; coloured outlines: short (light) to long (dark) timescales. **(B)** Same as above, but for word entropy.

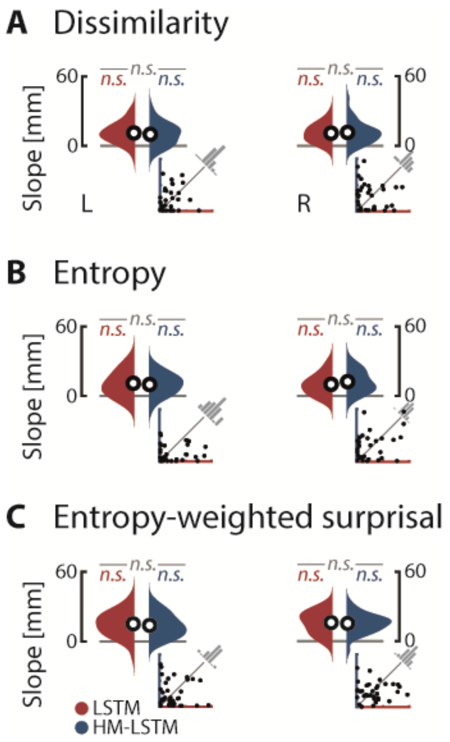

Figure S5. Testing for a processing hierarchy of secondary metrics of predictiveness. Along the dorsal stream, linear functions were fit to peak coordinates of **(A)** dissimilarity, **(B)** entropy and **(C)** entropy-weighted surprisal across timescales. Resulting slope parameters were compared to empirical null distributions (LSTM: red, HM-LSTM: blue) and between language models (LSTM vs. HM-LSTM: grey), separately for both hemispheres; black circles: grand-average slope parameters; insets: coefficients of determination for single-subject fits. *n.s.*: not significant.

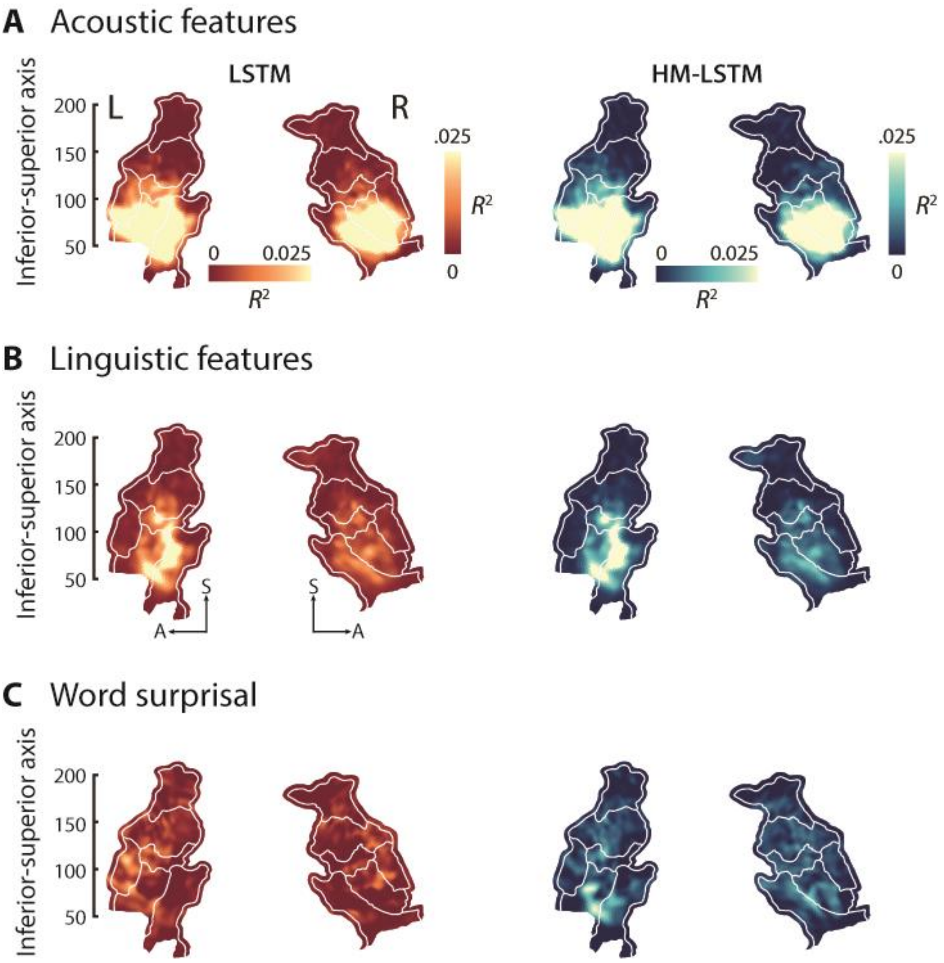

Figure S6. Variance uniquely explained by groups of regressors. (A) The temporo-parietal variance uniquely explained by acoustic features was calculated as the difference between the squared encoding accuracy of models including all groups of regressors and the squared encoding accuracy of models including scrambled acoustic regressors while keeping all other regressors intact, separately for both hemispheres and language models. **(B)** Maps of variance uniquely explained by linguistic features (i.e., word frequency, word length, content vs. function word). **(C)** Maps of variance uniquely explained by word surprisal (i.e., surprisal at single timescales and for full model).

REFERENCES AND NOTES

1. A. Clark, Whatever next? Predictive brains, situated agents, and the future of cognitive science. *Behav. Brain Sci.* **36**, 181–204 (2013).

2. J. D. Murray, A. Bernacchia, D. J. Freedman, R. Romo, J. D. Wallis, X. Cai, C. Padoa-Schioppa, T. Pasternak, H. Seo, D. Lee, X.-J. Wang, A hierarchy of intrinsic timescales across primate cortex. *Nat. Neurosci.* **17**, 1661–1663 (2014).

3. W. Zhang, M. M. Yartsev, Correlated neural activity across the brains of socially interacting bats. *Cell* **178**, 413–428.e22 (2019).

4. G. La Camera, A. Rauch, D. Thurbon, H.-R. Lüscher, W. Senn, S. Fusi, Multiple time scales of temporal response in pyramidal and fast spiking cortical neurons. *J. Neurophysiol.* **96**, 3448–3464 (2006).

5. J. B. Burt, M. Demirtaş, W. J. Eckner, N. M. Navejar, J. L. Ji, W. J. Martin, A. Bernacchia, A. Anticevic, J. D. Murray, Hierarchy of transcriptomic specialization across human cortex captured by structural neuroimaging topography. *Nat. Neurosci.* **21**, 1251–1259 (2018).

6. P. Lakatos, A. S. Shah, K. H. Knuth, I. Ulbert, G. Karmos, C. E. Schroeder, An oscillatory hierarchy controlling neuronal excitability and stimulus processing in the auditory cortex. *J. Neurophysiol.* **94**, 1904–1911 (2005).

7. M. G. Mattar, D. A. Kahn, S. L. Thompson-Schill, G. K. Aguirre, Varying timescales of stimulus integration unite neural adaptation and prototype formation. *Curr. Biol.* **26**, 1669–1676 (2016).

8. V. A. F. Lamme, P. R. Roelfsema, The distinct modes of vision offered by feedforward and recurrent processing. *Trends Neurosci.* **23**, 571–579 (2000).

9. U. Hasson, J. Chen, C. J. Honey, Hierarchical process memory: Memory as an integral component of information processing. *Trends Cogn. Sci.* **19**, 304–313 (2015).

10. G. T. Buračas, A. M. Zador, M. R. DeWeese, T. D. Albright, Efficient discrimination of temporal patterns by motion-sensitive neurons in primate visual cortex. *Neuron* **20**, 959–969 (1998).

11. C. A. Runyan, E. Piasini, S. Panzeri, C. D. Harvey, Distinct timescales of population coding across cortex. *Nature* **548**, 92–96 (2017).

12. R. Chaudhuri, K. Knoblauch, M.-A. Gariel, H. Kennedy, X.-J. Wang, A large-scale circuit mechanism for hierarchical dynamical processing in the primate cortex. *Neuron* **88**, 419–431 (2015).

13. M. Demirtaş, J. B. Burt, M. Helmer, J. L. Ji, B. D. Adkinson, M. F. Glasser, D. C. Van Essen, S. N. Sotiropoulos, A. Anticevic, J. D. Murray, Hierarchical heterogeneity across human cortex shapes large-scale neural dynamics. *Neuron* **101**, 1181–1194.e13 (2019).

14. J. M. Huntenburg, P.-L. Bazin, D. S. Margulies, Large-scale gradients in human cortical organization. *Trends Cogn. Sci.* **22**, 21–31 (2018).

15. K. D. Himberger, H.-Y. Chien, C. J. Honey, Principles of temporal processing across the cortical hierarchy. *Neuroscience* **389**, 161–174 (2018).

16. K. Friston, A theory of cortical responses. *Philos. Trans. R. Soc. B* **360**, 815–836 (2005).

17. G. B. Keller, T. D. Mrsic-Flogel, Predictive processing: A canonical cortical computation. *Neuron* **100**, 424–435 (2018).

18. S. J. Kiebel, J. Daunizeau, K. J. Friston, A hierarchy of time-scales and the brain. *PLOS Comput. Biol.* **4**, e1000209 (2008).

19. A. M. Bastos, J. Vezoli, C. A. Bosman, J.-M. Schoffelen, R. Oostenveld, J. R. Dowdall, P. De Weerd, H. Kennedy, P. Fries, Visual areas exert feedforward and feedback influences through distinct frequency channels. *Neuron* **85**, 390–401 (2015).

20. L. Cocchi, M. V. Sale, L. L. Gollo, P. T. Bell, V. T. Nguyen, A. Zalesky, M. Breakspear, J. B. Mattingley, A hierarchy of timescales explains distinct effects of local inhibition of primary visual cortex and frontal eye fields. *eLife* **5**, e15252 (2016).

21. C. Wacongne, E. Labyt, V. van Wassenhove, T. Bekinschtein, L. Naccache, S. Dehaene, Evidence for a hierarchy of predictions and prediction errors in human cortex. *Proc. Natl. Acad. Sci. U.S.A.* **108**, 20754–20759 (2011).

22. C. M. Schwiedrzik, W. A. Freiwald, High-level prediction signals in a low-level area of the macaque face-processing hierarchy. *Neuron* **96**, 89–97.e4 (2017).

23. P. W. Donhauser, S. Baillet, Two distinct neural timescales for predictive speech processing. *Neuron* **105**, 385–393.e9 (2020).

24. Z. C. Chao, K. Takaura, L. Wang, N. Fujii, S. Dehaene, Large-scale cortical networks for hierarchical prediction and prediction error in the primate brain. *Neuron* **100**, 1252–1266.e3 (2018).

25. G. J. Stephens, C. J. Honey, U. Hasson, A place for time: The spatiotemporal structure of neural dynamics during natural audition. *J. Neurophysiol.* **110**, 2019–2026 (2013).

26. W. A. de Heer, A. G. Huth, T. L. Griffiths, J. L. Gallant, F. E. Theunissen, The hierarchical cortical organization of human speech processing. *J. Neurosci.* **37**, 6539–6557 (2017).

27. Y. Lerner, C. J. Honey, L. J. Silbert, U. Hasson, Topographic mapping of a hierarchy of temporal receptive windows using a narrated story. *J. Neurosci.* **31**, 2906–2915 (2011).

28. C. H. C. Chang, C. Lazaridi, Y. Yeshurun, K. A. Norman, U. Hasson, Relating the past with the present: Information integration and segregation during ongoing narrative processing. *J. Cogn. Neurosci.* **33**, 1106–1128 (2021).

29. I. Bornkessel-Schlesewsky, M. Schlesewsky, S. L. Small, J. P. Rauschecker, Neurobiological roots of language in primate audition: Common computational properties. *Trends Cogn. Sci.* **19**, 142–150 (2015).

30. G. R. Kuperberg, T. F. Jaeger, What do we mean by prediction in language comprehension?, *Lang. Cogn. Neurosci.* **31**, 32–59 (2016).

31. L. H. Arnal, V. Wyart, A.-L. Giraud, Transitions in neural oscillations reflect prediction errors generated in audiovisual speech. *Nat. Neurosci.* **14**, 797–801 (2011).

32. H. Blank, M. H. Davis, Prediction errors but not sharpened signals simulate multivoxel fMRI patterns during speech perception. *PLOS Biol.* **14**, e1002577 (2016).

33. K. D. Kandylaki, A. Nagels, S. Tune, T. Kircher, R. Wiese, M. Schlesewsky, I. Bornkessel-Schlesewsky, Predicting "when" in discourse engages the human dorsal auditory stream: An fMRI study using naturalistic stories. *J. Neurosci.* **36**, 12180–12191 (2016).

34. K. Friston, S. Kiebel, Predictive coding under the free-energy principle. *Philos. Trans. R. Soc. B* **364**, 1211–1221 (2009).

35. J. Hale, A probabilistic earley parser as a psycholinguistic model, in *Proceedings of the 2nd North American Chapter of the Association for Computational Linguistics* (ACM Digital Library, 2001), pp. 1–8; http://portal.acm.org/citation.cfm?doid=1073336.1073357.

36. R. Levy, Expectation-based syntactic comprehension. *Cognition* **106**, 1126–1177 (2008).

37. R. M. Willems, S. L. Frank, A. D. Nijhof, P. Hagoort, A. van den Bosch, Prediction during natural language comprehension. *Cereb. Cortex* **26**, 2506–2516 (2016).

38. I. F. Monsalve, S. L. Frank, G. Vigliocco, Lexical surprisal as a general predictor of reading time, in *Proceedings of the 13th Conference of the European Chapter of the Association for Computational Linguistics* (Association for Computational Linguistics, 2012), pp. 398–408.

39. H.-Y. S. Chien, C. J. Honey, Constructing and forgetting temporal context in the human cerebral cortex. *Neuron* **106**, 675–686.e11 (2020).

40. A. Zadbood, J. Chen, Y. C. Leong, K. A. Norman, U. Hasson, How we transmit memories to other brains: Constructing shared neural representations via communication. *Cereb. Cortex* **27**, 4988–5000 (2017).

41. C. Baldassano, J. Chen, A. Zadbood, J. W. Pillow, U. Hasson, K. A. Norman, Discovering event structure in continuous narrative perception and memory. *Neuron* **95**, 709–721.e5 (2017).

42. J. Chung, S. Ahn, Y. Bengio, Hierarchical multiscale recurrent neural networks. arXiv:1609.01704v7 [cs.LG] (9 March 2017).

43. J. D. Cohen, N. Daw, B. Engelhardt, U. Hasson, K. Li, Y. Niv, K. A. Norman, J. Pillow, P. J. Ramadge, N. B. Turk-Browne, T. L. Willke, Computational approaches to fMRI analysis. *Nat. Neurosci.* **20**, 304–313 (2017).

44. R. M. Cichy, D. Kaiser, Deep neural networks as scientific models. *Trends Cogn. Sci.* **23**, 305–317 (2019).

45. S. Hochreiter, J. Schmidhuber, Long short-term memory. *Neural Comput.* **9**, 1735–1780 (1997).

46. S. Jain, A. Huth, Incorporating context into language encoding models for fMRI. bioRxiv 327601 [**Preprint**]. 21 May 2018. https://doi.org/10.1101/327601.

47. C. Aurnhammer, S. L. Frank, Evaluating information-theoretic measures of word prediction in naturalistic sentence reading. *Neuropsychologia* **134**, 107198 (2019).

48. J. Erb, L.-M. Schmitt, J. Obleser, Temporal selectivity declines in the aging human auditory cortex. *eLife* **9**, e55300 (2020).

49. S. A. Nastase, V. Gazzola, U. Hasson, C. Keysers, Measuring shared responses across subjects using intersubject correlation. *Soc. Cogn. Affect. Neurosci.* **14**, 667–685 (2019).

50. J. R. Binder, R. H. Desai, The neurobiology of semantic memory. *Trends Cogn. Sci.* **15**, 527–536 (2011).

51. M. F. Glasser, T. S. Coalson, E. C. Robinson, C. D. Hacker, J. Harwell, E. Yacoub, K. Ugurbil, J. Andersson, C. F. Beckmann, M. Jenkinson, S. M. Smith, D. C. Van Essen, A multi-modal parcellation of human cerebral cortex. *Nature* **536**, 171–178 (2016).

52. K. J. Friston, C. Buechel, G. R. Fink, J. Morris, E. Rolls, R. J. Dolan, Psychophysiological and modulatory interactions in neuroimaging. *Neuroimage* **6**, 218–229 (1997).

53. U. Hasson, E. Yang, I. Vallines, D. J. Heeger, N. Rubin, A hierarchy of temporal receptive windows in human cortex. *J. Neurosci.* **28**, 2539–2550 (2008).

54. J. M. Zacks, N. K. Speer, K. M. Swallow, T. S. Braver, J. R. Reynolds, Event perception: A mind-brain perspective. *Psychol. Bull.* **133**, 273–293 (2007).

55. G. A. Radvansky, Across the event horizon. *Curr. Dir. Psychol. Sci.* **21**, 269–272 (2012).

56. J. M. Zacks, T. S. Braver, M. A. Sheridan, D. I. Donaldson, A. Z. Snyder, J. M. Ollinger, R. L. Buckner, M. E. Raichle, Human brain activity time-locked to perceptual event boundaries. *Nat. Neurosci.* **4**, 651–655 (2001).

57. C. Whitney, W. Huber, J. Klann, S. Weis, S. Krach, T. Kircher, Neural correlates of narrative shifts during auditory story comprehension. *Neuroimage* **47**, 360–366 (2009).

58. L. L. Richmond, J. M. Zacks, Constructing experience: Event models from perception to action. *Trends Cogn. Sci.* **21**, 962–980 (2017).

59. F. Lieder, K. E. Stephan, J. Daunizeau, M. I. Garrido, K. J. Friston, A neurocomputational model of the mismatch negativity. *PLOS Comput. Biol.* **9**, e1003288 (2013).

60. A. Todorovic, F. P. de Lange, Repetition suppression and expectation suppression are dissociable in time in early auditory evoked fields. *J. Neurosci.* **32**, 13389–13395 (2012).

61. P. Hagoort, G. Baggio, R. M. Willems, Semantic unification, in *The Cognitive Neurosciences* (MIT Press, ed. 4, 2009), pp. 819–836.

62. P. Kok, J. F. M. Jehee, F. P. de Lange, Less is more: Expectation sharpens representations in the primary visual cortex. *Neuron* **75**, 265–270 (2012).

63. R. P. N. Rao, D. H. Ballard, Predictive coding in the visual cortex: A functional interpretation of some extra-classical receptive-field effects. *Nat. Neurosci.* **2**, 79–87 (1999).

64. G. Hickok, D. Poeppel, The cortical organization of speech processing. *Nat. Rev. Neurosci.* **8**, 393–402 (2007).

65. I. DeWitt, J. P. Rauschecker, Phoneme and word recognition in the auditory ventral stream. *Proc. Natl. Acad. Sci. U.S.A.* **109**, E505–E514 (2012).

66. I. Bornkessel-Schlesewsky, M. Schlesewsky, Reconciling time, space and function: A new dorsal–ventral stream model of sentence comprehension. *Brain Lang.* **125**, 60–76 (2013).

67. S. M. Wilson, I. Molnar-Szakacs, M. Iacoboni, Beyond superior temporal cortex: Intersubject correlations in narrative speech comprehension. *Cereb. Cortex* **18**, 230–242 (2008).

68. H. N. Phillips, A. Blenkmann, L. E. Hughes, S. Kochen, T. A. Bekinschtein, Cam-CAN, J. B. Rowe, Convergent evidence for hierarchical prediction networks from human electrocorticography and magnetoencephalography. *Cortex* **82**, 192–205 (2016).

69. B. Lyu, J. Ge, Z. Niu, L. H. Tan, J.-H. Gao, Predictive brain mechanisms in sound-to-meaning mapping during speech processing. *J. Neurosci.* **36**, 10813–10822 (2016).

70. F. Meyniel, S. Dehaene, Brain networks for confidence weighting and hierarchical inference during probabilistic learning. *Proc. Natl. Acad. Sci. U.S.A.* **114**, E3859–E3868 (2017).

71. V. K. M. Cheung, L. Meyer, A. D. Friederici, S. Koelsch, The right inferior frontal gyrus processes nested non-local dependencies in music. *Sci. Rep.* **8**, 3822 (2018).

72. L. H. Tan, A. R. Laird, K. Li, P. T. Fox, Neuroanatomical correlates of phonological processing of Chinese characters and alphabetic words: A meta-analysis. *Hum. Brain Mapp.* **25**, 83–91 (2005).

73. D. Ayyash, S. Malik-Moraleda, J. Gallée, J. Affourtit, M. Hoffman, Z. Mineroff, O. Jouravlev, E. Fedorenko, The universal language network: A cross-linguistic investigation spanning 45 languages and 11 language families. bioRxiv 2021.07.28.454040 [**Preprint**]. 29 July 2021. https://doi.org/10.1101/2021.07.28.454040.

74. R. Bottini, C. F. Doeller, Knowledge across reference frames: Cognitive maps and image spaces. *Trends Cogn. Sci.* **24**, 606–619 (2020).

75. I. K. Brunec, M. Moscovitch, M. D. Barense, Boundaries shape cognitive representations of spaces and events. *Trends Cogn. Sci.* **22**, 637–650 (2018).

76. A. S. Alexander, D. A. Nitz, Spatially periodic activation patterns of retrosplenial cortex encode route sub-spaces and distance traveled. *Curr. Biol.* **27**, 1551–1560.e4 (2017).

77. K. L. Stachenfeld, M. M. Botvinick, S. J. Gershman, The hippocampus as a predictive map. *Nat. Neurosci.* **20**, 1643–1653 (2017).

78. J. D. Power, K. A. Barnes, A. Z. Snyder, B. L. Schlaggar, S. E. Petersen, Spurious but systematic correlations in functional connectivity MRI networks arise from subject motion. *Neuroimage* **59**, 2142–2154 (2012).

79. A. U. Rysop, L.-M. Schmitt, J. Obleser, G. Hartwigsen, Neural modelling of the semantic predictability gain under challenging listening conditions. *Hum. Brain Mapp.* **42**, 110–127 (2021).

80. J. H. McDermott, E. P. Simoncelli, Sound texture perception via statistics of the auditory periphery: evidence from sound synthesis. *Neuron* **71**, 926–940 (2011).

81. T. Kisler, U. Reichel, F. Schiel, Multilingual processing of speech via web services. *Comput. Speech Lang.* **45**, 326–347 (2017).

82. M. Brysbaert, M. Buchmeier, M. Conrad, A. M. Jacobs, J. Bölte, A. Böhl, The word frequency effect: A review of recent developments and implications for the choice of frequency estimates in German. *Exp. Psychol.* **58**, 412–424 (2011).

83. W. J. B. van Heuven, P. Mandera, E. Keuleers, M. Brysbaert, Subtlex-UK: A new and improved word frequency database for British English. *Q. J. Exp. Psychol.* **67**, 1176–1190 (2014).

84. D. H. Brainard, The Psychophysics Toolbox. *Spat. Vis.* **10**, 433–436 (1997).

85. B. Heinzerling, M. Strube, BPEmb: Tokenization-free pre-trained subword embeddings in 275 languages. arXiv:1710.02187 [cs] (5 October 2017).

86. Á. Kádár, M.-A. Côté, G. Chrupała, A. Alishahi, Revisiting the hierarchical multiscale LSTM. arXiv:1807.03595 [cs] (10 July 2018).

87. D. P. Kingma, J. Ba, Adam: A method for stochastic optimization. arXiv:1412.6980 [cs] (30 December 2017).

88. A. Barbaresi, A corpus of German political speeches from the 21st century, in *11th Language Resources and Evaluation Conference* (ELRA, 2018), pp. 792–797.

89. M. P. Broderick, A. J. Anderson, G. M. Di Liberto, M. J. Crosse, E. C. Lalor, Electrophysiological correlates of semantic dissimilarity reflect the comprehension of natural, narrative speech. *Curr. Biol.* **28**, 803–809.e3 (2018).

90. T. Chi, P. Ru, S. A. Shamma, Multiresolution spectrotemporal analysis of complex sounds. *J. Acoust. Soc. Am.* **118**, 887–906 (2005).

91. M. Kumar, C. T. Ellis, Q. Lu, H. Zhang, M. Capotă, T. L. Willke, P. J. Ramadge, N. B. Turk-Browne, K. A. Norman, BrainIAK tutorials: User-friendly learning materials for advanced fMRI analysis. *PLOS Comput. Biol.* **16**, e1007549 (2020).

92. Y. Benjamini, Y. Hochberg, Controlling the false discovery rate: A practical and powerful approach to multiple testing. *J. R. Stat. Soc. Ser. B Methodol.* **57**, 289–300 (1995).

93. R. Santoro, M. Moerel, F. De Martino, G. Valente, K. Ugurbil, E. Yacoub, E. Formisano, Reconstructing the spectrotemporal modulations of real-life sounds from fMRI response patterns. *Proc. Natl. Acad. Sci. U.S.A.* **114**, 4799–4804 (2017).

94. G. H. Golub, M. Heath, G. Wahba, Generalized cross-validation as a method for choosing a good ridge parameter. *Technometrics* **21**, 215–223 (1979).

95. S. Musall, M. T. Kaufman, A. L. Juavinett, S. Gluf, A. K. Churchland, Single-trial neural dynamics are dominated by richly varied movements. *Nat. Neurosci.* **22**, 1677–1686 (2019).

96. F. Pedregosa, G. Varoquaux, A. Gramfort, V. Michel, B. Thirion, O. Grisel, M. Blondel, P. Prettenhofer, R. Weiss, V. Dubourg, J. Vanderplas, A. Passos, D. Cournapeau, Scikit-learn: Machine learning in Python. *J. Mach. Learn. Res.* **12**, 2825–2830 (2011).

97. E. Maris, R. Oostenveld, Nonparametric statistical testing of EEG- and MEG-data. *J. Neurosci. Methods* **164**, 177–190 (2007).

98. D. C. Mitchell, An evaluation of subject-paced reading tasks and other methods for investigating immediate processes in reading, in *New Methods in Reading Comprehension Research* (Erlbaum, 1984), pp. 69–89.

99. N. J. Smith, R. Levy, The effect of word predictability on reading time is logarithmic. *Cognition* **128**, 302–319 (2013).

100. K. Rayner, Eye movements in reading and information processing: 20 years of research. *Psychol. Bull.* **124**, 372–422 (1998).

101. J. Pennington, R. Socher, C. Manning, GloVe: Global vectors for word representation, in *Proceedings of the 2014 Conference on Empirical Methods in Natural Language Processing* (Association for Computational Linguistics, 2014), pp. 1532–1543; http://aclweb.org/anthology/D14-1162.

102. J. L. Ba, J. R. Kiros, G. E. Hinton, Layer normalization. arXiv:1607.06450 [cs, stat] (21 July 2016).

103. W. Penny, K. Friston, J. Ashburner, S. Kiebel, T. Nichols, *Statistical Parametric Mapping: The Analysis of Functional Brain Images* (Academic Press, 2006).

104. O. Esteban, C. J. Markiewicz, R. W. Blair, C. A. Moodie, A. I. Isik, A. Erramuzpe, J. D. Kent, M. Goncalves, E. DuPre, M. Snyder, H. Oya, S. S. Ghosh, J. Wright, J. Durnez, R. A. Poldrack, K. J. Gorgolewski, fMRIPrep: A robust preprocessing pipeline for functional MRI. *Nat. Methods* **16**, 111–116 (2019).

105. K. Gorgolewski, C. D. Burns, C. Madison, D. Clark, Y. O. Halchenko, M. L. Waskom, S. S. Ghosh, Nipype: A flexible, lightweight and extensible neuroimaging data processing framework in Python. *Front. Neuroinform.* **5**, 13 (2011).

106. A. Abraham, F. Pedregosa, M. Eickenberg, P. Gervais, A. Mueller, J. Kossaifi, A. Gramfort, B. Thirion, G. Varoquaux, Machine learning for neuroimaging with scikit-learn. *Front. Neuroinform.* **8**, 14 (2014).

107. N. J. Tustison, B. B. Avants, P. A. Cook, Y. Zheng, A. Egan, P. A. Yushkevich, J. C. Gee, N4ITK: Improved N3 bias correction. *IEEE Trans. Med. Imaging* **29**, 1310–1320 (2010).

108. A. M. Dale, B. Fischl, M. I. Sereno, Cortical surface-based analysis. I. Segmentation and surface reconstruction. *Neuroimage* **9**, 179–194 (1999).

109. V. Fonov, A. Evans, R. McKinstry, C. Almli, D. Collins, Unbiased nonlinear average age-appropriate brain templates from birth to adulthood. *Neuroimage* **47**, S102 (2009).

110. B. Avants, C. Epstein, M. Grossman, J. Gee, Symmetric diffeomorphic image registration with cross-correlation: Evaluating automated labeling of elderly and neurodegenerative brain. *Med. Image Anal.* **12**, 26–41 (2008).

111. A. Klein, S. S. Ghosh, F. S. Bao, J. Giard, Y. Häme, E. Stavsky, N. Lee, B. Rossa, M. Reuter, E. Chaibub Neto, A. Keshavan, Mindboggling morphometry of human brains. *PLOS Comput. Biol.* **13**, e1005350 (2017).

112. Y. Zhang, M. Brady, S. Smith, Segmentation of brain MR images through a hidden Markov random field model and the expectation-maximization algorithm. *IEEE Trans. Med. Imaging* **20**, 45–57 (2001).

113. M. Jenkinson, P. Bannister, M. Brady, S. Smith, Improved optimization for the robust and accurate linear registration and motion correction of brain images. *Neuroimage* **17**, 825–841 (2002).

114. R. W. Cox, AFNI: Software for analysis and visualization of functional magnetic resonance neuroimages. *Comput. Biomed. Res.* **29**, 162–173 (1996).

115. D. N. Greve, B. Fischl, Accurate and robust brain image alignment using boundary-based registration. *Neuroimage* **48**, 63–72 (2009).

116. R. H. R. Pruim, M. Mennes, D. van Rooij, A. Llera, J. K. Buitelaar, C. F. Beckmann, ICA-AROMA: A robust ICA-based strategy for removing motion artifacts from fMRI data. *Neuroimage* **112**, 267–277 (2015).

117. J. D. Power, A. Mitra, T. O. Laumann, A. Z. Snyder, B. L. Schlaggar, S. E. Petersen, Methods to detect, characterize, and remove motion artifact in resting state fMRI. *Neuroimage* **84**, 320–341 (2014).

118. M. A. Lindquist, S. Geuter, T. D. Wager, B. S. Caffo, Modular preprocessing pipelines can reintroduce artifacts into fMRI data. *Hum. Brain Mapp.* **40**, 2358–2376 (2019).

119. J. S. Guntupalli, M. Hanke, Y. O. Halchenko, A. C. Connolly, P. J. Ramadge, J. V. Haxby, A model of representational spaces in human cortex. *Cereb. Cortex* **26**, 2919–2934 (2016).

120. M. Hanke, Y. O. Halchenko, P. B. Sederberg, S. J. Hanson, J. V. Haxby, S. Pollmann, PyMVPA: a Python toolbox for multivariate pattern analysis of fMRI data. *Neuroinformatics* **7**, 37–53 (2009).

121. R. Boldt, S. Malinen, M. Seppä, P. Tikka, P. Savolainen, R. Hari, S. Carlson, Listening to an audio drama activates two processing networks, one for all sounds, another exclusively for speech. *PLOS ONE* **8**, e64489 (2013).

122. R. Schmälzle, F. E. K. Häcker, C. J. Honey, U. Hasson, Engaged listeners: Shared neural processing of powerful political speeches. *Soc. Cogn. Affect. Neurosci.* **10**, 1137–1143 (2015).

123. M. Regev, E. Simony, K. Lee, K. M. Tan, J. Chen, U. Hasson, Propagation of information along the cortical hierarchy as a function of attention while reading and listening to stories. *Cereb. Cortex* **29**, 4017–4034 (2018).
